# Cardiac involvement in patient-specific induced pluripotent stem cells of myotonic dystrophy type 1: unveiling the impact of voltage-gated sodium channels

**DOI:** 10.3389/fphys.2023.1258318

**Published:** 2023-09-18

**Authors:** Marion Pierre, Mohammed Djemai, Charles-Albert Chapotte-Baldacci, Valérie Pouliot, Jack Puymirat, Mohamed Boutjdir, Mohamed Chahine

**Affiliations:** ^1^ CERVO Research Center, Quebec City, QC, Canada; ^2^ LOEX, CHU de Québec-Université Laval Research Center, Quebec City, QC, Canada; ^3^ Cardiovascular Research Program, VA New York Harbor Healthcare System, Brooklyn, NY, United States; ^4^ Departments of Cell Biology and Pharmacology, SUNY Downstate Health Sciences University, Brooklyn, NY, United States; ^5^ Department of Medicine, New York University Grossman School of Medicine, New York, NY, United States; ^6^ Department of Medicine, Faculty of Medicine, Université Laval, Quebec City, QC, Canada

**Keywords:** myotonic dystrophy type 1, atrial hiPSC-CMs, ventricular hiPSC-CMs, Na_V_1.5, optical mapping, cardiac arrhythmias, conduction

## Abstract

Myotonic dystrophy type 1 (DM1) is a genetic disorder that causes muscle weakness and myotonia. In DM1 patients, cardiac electrical manifestations include conduction defects and atrial fibrillation. DM1 results in the expansion of a CTG transcribed into CUG-containing transcripts that accumulate in the nucleus as RNA foci and alter the activity of several splicing regulators. The underlying pathological mechanism involves two key RNA-binding proteins (MBNL and CELF) with expanded CUG repeats that sequester MBNL and alter the activity of CELF resulting in spliceopathy and abnormal electrical activity. In the present study, we identified two DM1 patients with heart conduction abnormalities and characterized their hiPSC lines. Two differentiation protocols were used to investigate both the ventricular and the atrial electrophysiological aspects of DM1 and unveil the impact of the mutation on voltage-gated ion channels, electrical activity, and calcium homeostasis in DM1 cardiomyocytes derived from hiPSCs. Our analysis revealed the presence of molecular hallmarks of DM1, including the accumulation of RNA foci and sequestration of MBNL1 in DM1 hiPSC-CMs. We also observed mis-splicing of *SCN5A* and haploinsufficiency of DMPK. Furthermore, we conducted separate characterizations of atrial and ventricular electrical activity, conduction properties, and calcium homeostasis. Both DM1 cell lines exhibited reduced density of sodium and calcium currents, prolonged action potential duration, slower conduction velocity, and impaired calcium transient propagation in both ventricular and atrial cardiomyocytes. Notably, arrhythmogenic events were recorded, including both ventricular and atrial arrhythmias were observed in the two DM1 cell lines. These findings enhance our comprehension of the molecular mechanisms underlying DM1 and provide valuable insights into the pathophysiology of ventricular and atrial involvement.

## 1 Introduction

Myotonic dystrophy type 1 (DM1), also known as Steinert’s disease, is a multisystemic genetic disorder characterized by progressive muscle weakness and myotonia, which affects approximately 1 in 8,000 individuals worldwide (Ozimski et al., 2021). It is well known that the pathogenesis is multifactorial and involves an RNA gain-of-function. Dystrophia Myotonica Protein Kinase (*DMPK*) repeat-containing transcripts form nuclear aggregates called foci, leading to altered splicing patterns, and disrupted cellular functions (Wheeler and Thornton, 2007). Two key RNA-binding protein families, MBNL (muscleblind-like) and CELF (CUG-BP and ETR-3-like factor), are implicated in DM1. Expanded CUG repeats sequester MBNL and cause a loss of its function, contributing to cardiac spliceopathy and electrical abnormalities in DM1, as also observed in Mbnl1-deficient mouse models. (Lee et al., 2013; Dixon et al., 2015; Lee et al., 2022). CELF1 upregulation due to abnormal PKC signaling has also been observed in a DM1 mouse model with dilated cardiomyopathy and arrhythmia (Wang et al., 2009). A prominent splicing defect involves *SCN5A*, which encodes the cardiac sodium channel, Na_V_1.5, leading to conduction abnormalities (Freyermuth et al., 2016). Aberrant splicing of *SCN5A* mRNA switches the adult isoform to the fetal isoform, resulting in lower excitability (Onkal et al., 2008). Mouse models with the fetal *Scn5a* isoform exhibit conduction system disorders (Freyermuth et al., 2016; Pang et al., 2018).

Cardiac manifestations are observed in approximately 80% of DM1 patients, including conduction abnormalities, atrial and ventricular arrhythmias, and structural abnormalities (Russo et al., 2023). Conduction defects, such as first degree atrioventricular block (AV block), are frequently reported (Mahadevan et al., 2021). Other ECG abnormalities, including bundle branch blocks and prolongation of the QRS interval, are also common. Atrial fibrillation and flutter have an estimated prevalence of 11% and 8.5%, respectively (Wahbi et al., 2016; Russo et al., 2021). Structural abnormalities such as left ventricular hypertrophy contribute to cardiac dysfunction (Wahbi and Furling, 2020; Mahadevan et al., 2021; Russo et al., 2023). Sudden cardiac death is one of the most frequent causes of death in DM1 (43% of patients) (Wahbi et al., 2018). Studies have consistently demonstrated that the severity of cardiac involvement is correlated with the size of the CTG repeats (Chong-Nguyen et al., 2017).

Human induced pluripotent stem cells (hiPSCs) have become a valuable tool for disease modeling and have allowed the generation of hiPSC-derived cardiomyocytes (hiPSC-CMs) that mimic cardiac disease characteristics. Various studies, including ours, have successfully replicated key features of DM1 cardiac pathogenesis using hiPSC-CMs (Veerman et al., 2017; Spitalieri et al., 2018; Kim et al., 2019; Poulin et al., 2021; Dastidar et al., 2022). Notably, our prior research highlighted two major perturbations in ion channels in DM1, one affecting sodium channels and the other affecting calcium channels (Poulin et al., 2021).

In the present study, we aimed to expand our investigation to encompass additional DM1 patients. As such, we identified two new DM1 patients with heart conduction abnormalities, 55-year-old (DM1-1290) and 30-year-old (DM1-1640) male patients with 1291 and 1639 CTG repeats, respectively whose hiPSC lines have recently been characterized (Jauvin et al., 2023). Two distinct differentiation protocols were used, *for the first time*, to investigate both ventricular (vCMs) and atrial (aCMs) aspects in the context of DM1. We studied the molecular hallmarks of DM1 and their impact on atrial and ventricular electrical activity, conduction, and calcium homeostasis.

## 2 Materials and methods

### 2.1 Patient-specific hiPSC description

The hiPSC lines were derived from Epstein-Barr virus (EBV) immortalized lymphoblastoid cell lines (LCL) and were reprogrammed at the LOEX core facility in Quebec City, Canada. We characterized the hiPSC lines (Jauvin et al., 2023) using an assessment of CTG repeat lengths by Southern blot analyses, an evaluation of pluripotency-associated protein expression, an examination of pluripotency marker gene expression, a determination of the differentiation potential into three germ layers, a verification of the identity of the hiPSCs compared to the original cells, and an analysis of the karyotypes. We studied two specific hiPSC lines: CBRCULi002-A, which was derived from a 55-year-old male patient with 1291 CTG repeats (hereinafter referred to as DM1-1290), and CBRCULi004-A, which was derived from a 30-year-old male patient with 1639 CTG repeats (hereinafter referred to as DM1-1640). These DM1 lines were compared to a control healthy hiPSC line from a 44-year-old male (CBRCULi001-A, referred to as CTRL). The study protocol was approved by the local ethics committee (Project #2019-1734).

### 2.2 hiPSC culture and cardiomyocyte differentiation

The hiPSCs were cultured on hESC-qualified Matrigel (Cat# 354277, Corning, AZ, United States) in mTeSR plus medium (Cat# 100-0276, STEMCELL Technologies, BC, Canada), and were regularly passaged every 4–6 days using 500 μM EDTA. They were then induced to differentiate into ventricular cardiomyocytes (vCMs) using STEMdiff™ Ventricular Cardiomyocyte Differentiation kits (Cat# 05010, STEMCELL Technologies) according to the manufacturer’s protocols and instructions. The hiPSCs were also differentiated into atrial cardiomyocytes (aCMs) by applying all-trans retinoic acid (RA) between 2 and 5 days (Cat# 72262, STEMCELL Technologies) during the differentiation process. Spontaneously beating cells were observed on day 8–12 of differentiation. All experiments were conducted on day 30 of maturation. We exclusively chose spontaneously beating monolayers that exhibited efficient differentiation to perform the experiments.

### 2.3 Fluorescence *in situ* hybridization (FISH) and immunocytofluorescence staining

The hiPSC-CMs were dissociated using STEMdiff™ Cardiomyocyte Dissociation Medium (Cat# 05025, STEMCELL Technologies) on day 23 and were plated on Matrigel-coated 12-mm glass coverslips in a 24-well plate at a density of 50,000 cells/cm^2^. The cells were fixed with 4% paraformaldehyde (PFA, Cat# 15710, Electron Microscopy Sciences), permeabilized in a PBS (phosphate buffered saline) solution containing 0.2% Triton X-100, 5% goat serum, and 1% BSA for 30 min on day 30. The hiPSC-CMs were then stained overnight at 4°C using a blocking solution (1% BSA/5% GS in PBS) containing the following primary antibodies: mouse anti-troponin T2, cardiac type (TNNT2, 1:300, Cat# ab10214, Abcam, RRID: AB 2206574), rabbit anti-muscleblind like splicing regulator 1 (MBNL1, 1/250, Cat# NBP2-55165, Novus Biologicals), rabbit anti-gap junction protein alpha 1 (GJA1/Cx43, 1:200, Cat# ab11370, Abcam, RRID: AB_297976), and mouse anti-actinin alpha 2 (ACTN2, 1:500, Cat# ab9465, Abcam, RRID: AB_307264). After washing, the secondary antibodies [goat anti-mouse AlexaFluor™ 488 (1:2000, Cat# A21141, Invitrogen, RRID: AB_2535778), goat anti-rabbit AlexaFluor™ 633 (1:2000, Cat# A21071, Invitrogen, RRID: AB_2535732), and goat anti-mouse Cy3 (1:500, Cat# A10521, Invitrogen, RRID:AB_2534030)] were then added to the blocking solution. The mixtures were incubated for 2 h at room temperature in the dark. After washing, the cells were fixed again with 4% PFA and were washed with 2X SSC (sodium chloride/sodium citrate)/50% formamide. They were then incubated with the hybridization solution [1 µg/uL of yeast tRNA extract, 5% dextran sulfate, 0.3% BSA, 50% formamide, 2X SSC, 2 mM vanadyl ribonucleoside complex, and 1 ng/uL of 5′-Cy3-labeled (CAG)_5_ peptide nucleotide probe (PNA Bio, Cat# F5001)] for 2 h at 37°C in the dark. The cells were washed twice with 2X SSC/50% formamide for 30 min at 37°C in the dark, and were mounted on slides using Fluoromount-G mounting medium with DAPI (Cat# 00-4959-52, Invitrogen). The immunolabeled samples were observed using a Zeiss LSM780 confocal laser scanning microscope (Zeiss, Germany).

### 2.4 Gene expression analysis

qPCR assays were performed on day 30 of maturation to analyze hiPSC-CM gene expressions. The RNA was extracted from the hiPSC-CMs using quick-RNA MiniPrep kits (Cat# R1054, Cedarlane, ON, Canada), and the cDNA synthesis was performed using the QuantiTect Rev. Transcription Kit protocol (Cat# 205313, Qiagen, Hilden, Germany). qPCR assays were performed on an LC480 platform (Roche, Basel, Switzerland) using SYBR green I detection dye following the specifications provided by the manufacturer. The qPCR reactions were run in triplicate, accompanied by a non-template control (NTC). The determination of qPCR efficiencies involved the utilization of a sequence of cDNA dilutions, with calculations executed based on the slope of the regression line derived from the subsequent equation: E = 10 [–1/slope]. The range of efficiency for all qPCR reactions spanned from 1.7 to 2.1. Analysis was carried out through employment of LightCycler^®^ 480 SW 1.5 software. Adjustment for run-to-run variabilities was achieved through the utilization of a recognized standard, and quantification was rectified for efficiency variations. To validate the specificity of the amplification in each run, a melting curve analysis was employed. Normalization was performed using three housekeeping genes (GATA binding protein 4 (*GATA4*), ribosomal protein L22 (*RPL22*)*,* and peptidylprolyl isomerase A (*PPIA*)). For the analysis of *SCN5A* mRNA, exon 25 of *SCN5A* mRNA encompassed all the isoforms, including the fetal (exon 6a) and adult (exon 6b) sodium channel isoforms. The primers can be found in Supplementary Table S1.

### 2.5 Western blotting

On day 30 of maturation, the total proteins were extracted from the hiPSC-CMs by gently scraping the cells into Radioimmunoprecipitation Assay buffer (RIPA buffer: 50 mmol/L Tris-Cl, 1 mmol/L EDTA, 150 mmol/L NaCl, 0.5% SDS, 1% NP-40) supplemented with proteases (Cat# 5892970001, Sigma-Aldrich) and phosphatase (Cat# 4906845001, Sigma-Aldrich) inhibitor cocktails. The resulting lysate was incubated for 2 h at 4°C under gentle rotation, followed by clarification through centrifugation at 18,000 g for 5 min at 4°C. Measurement of total protein concentrations was conducted using Pierce™ BCA Protein Assay kits (Cat# 23225, ThermoFisher Scientific) with a bovine serum albumin (BSA) standard range (20–2000 μg/mL) as a reference. Protein extracts (15 μg) were denatured in ×5 sample buffer (156 mM Tris-Cl, pH 6.8, 0.025% bromophenol blue, 5% SDS, 50% glycerol, 12.5% β-mercaptoethanol) at 37°C for 30 min. They were resolved on 4%–15% Mini-PROTEAN^®^ TGX Stain-Free™ Protein gels (Cat# 456-8083, Bio-Rad), and were blotted on 0.45-μm PVDF membranes with Trans-Blot Turbo RTA Midi 0.45 µm LF PVDF Transfer kits (Cat# 1704275, BioRad). The PVDF membranes were blocked and were incubated with the following primary antibodies: rabbit anti-SCN5A (Na_V_1.5, 1:200, Cat# ASC-005, Alomone Labs, RRID: AB_2040001), rabbit anti-calcium voltage-gated channel subunit alpha 1 C (CACNA1C/Ca_V_1.2, 1:200, Cat# ACC-003, Alomone Labs, RRID:AB_2039771), rabbit anti-MBNL1 (1/500, Cat# NBP2-55165, Novus Biologicals), rabbit anti-GJA1 (Cx43, 1:5000, Cat# ab11370, Abcam, RRID: AB_297976), mouse anti-CELF1 (1:1000, Cat# SC-20003, Santa Cruz Biotechnology, RRID: AB_627319), and mouse anti-DMPK (1:750, cat# SC-134319, Santa Cruz Biotechnology, RRID: AB_2091375). After washing, the following secondary antibodies were added: goat horseradish peroxidase (HRP)-conjugated anti-rabbit (1:10,000, Cat# 111-035-003, Jackson ImmunoResearch, RRID: AB_2313567), and anti-mouse (1:10,000, Cat# 115-035-003, Jackson ImmunoResearch, RRID: AB_10015289). The proteins were visualized using the ChemiDoc system (Bio-Rad, ON, Canada) after being revealed with the Clarity and Clarity Max Western ECL substrates (Cat# 1705060 and Cat# 1705062, Bio-Rad). ImageJ software was used for the relative quantification of protein expression. To normalize the intensities of the targeted protein bands, the total intensities of the bands were divided by the normalization factor, which was determined by dividing the observed signals of total protein in the lane with the strongest observed signal of total protein on the blot. Pictures of gels and blots can be found in Supplementary Figure S1.

### 2.6 Electrophysiology

On day 30 of maturation, the macroscopic sodium current, I_Na_ and L-type calcium current, I_CaL_ as well as action potentials (APs) were measured using the whole-cell configuration of the patch-clamp technique in the voltage-clamp and current-clamp modes, respectively, as previously described (Pierre et al., 2021). Briefly, the experiments were performed at room temperature using an Axopatch 200B amplifier and pClamp software v10 (Molecular Devices, CA, United States). The pipettes were drawn from borosilicate glass capillaries (Sutter Instrument, CA, United States), and were fire polished. The pipettes are gently brought into proximity with the cellular membrane using a motorized PatchStar micromanipulator (Scientifica, CA, United States). By applying controlled suction, a tight seal forms between the pipette tip and the membrane, creating a high-resistance junction. With increased suction, the pipette ruptures a small portion of the membrane, establishing whole-cell configuration.

For the voltage-clamp experiments, the pipettes were coated with HIPEC (Dow-Corning, MI, United States) to minimize electrode capacitance. For the I_Na_ currents, the pipettes were filled with a solution containing (in mmol/L): 35 NaCl, 105 CsF, 10 EGTA, and 10 HEPES. The pH was adjusted to 7.4 with CsOH. The bath solution contained (in mmol/L): 105 NMDG, 35 NaCl, 2 KCl, 1.5 CaCl_2_, 1 MgCl_2_, 10 D-glucose, 10 HEPES, 10 TEA-Cl, and 0.01 nifedipine to inhibit I_Ca_. The pH was adjusted to 7.4 with methanethiosulfonic (MTS) acid. For the I_Ca_ currents, the pipettes were filled with a solution containing (in mmol/L): 25 NaCl, 105 CsCl, 1 MgCl_2_, 10 EGTA, and 10 HEPES. The pH was adjusted to 7.2 with CsOH. The bath solution contained (in mmol/L): 100 NaCl, 5 CsCl, 5 CaCl_2_, 40 NMDG, 1 MgCl_2_, 10 D-glucose, 10 HEPES, and 15 TEA-Cl. The pH was adjusted to 7.4 with methane sulfonic acid (MSA). Series resistance and cell capacitance were compensated. Currents were filtered at 5 kHz, digitized at 10 kHz, and stored on a microcomputer outfitted with an AD converter (Digidata 1440A, Molecular Devices). P/4 leak subtraction was implemented prior to the application of pulse stimulations for sodium and calcium current recordings.

For the current-clamp experiments, the patch pipettes (with a resistance of 2–5 MΩ) were filled with a solution containing (in mmol/L): 10 NaCl, 122 KCl, 1 MgCl_2_, 1 EGTA, and 10 HEPES. The pH was adjusted to 7.3 with KOH. The bath solution was composed of (in mmol/L): 154 NaCl, 5.6 KCl, 2 CaCl_2_, 1 MgCl_2_, 8 D-glucose, and 10 HEPES. The pH was adjusted to 7.3 with NaOH. The properties of the APs were studied using different stimulation frequencies (0.5, 1.0, 1.5, and 2.0 Hz) while maintaining a holding potential of −80 mV. To do that, we imposed −80 mV by using the holding command mode of the amplifier device, which creates a constant current injection. This approach induces the closure of certain ion channels, which resulted in the generation of well-defined APs with significant amplitudes. This technique also facilitated the stabilization of the cells and suppressed spontaneous activity. A 3-ms stimulation pulse was used and was accompanied by the injection of varying currents, named thresholds of depolarization. Prior to establishing the holding potential of −80 mV and initiating the stimulation, the resting membrane potential (RMP) was measured. The spontaneous APs were recorded using the gap-free mode following the stimulation.

### 2.7 Optical mapping

hiPSC-CMs in monolayer were generated by seeding hiPSC-CMs onto hESC-qualified Matrigel-coated 12-mm TC coverslips in a 24-well plate between days 12 and 15. Briefly, at day 30, the monolayers were stained with Rhod-2 AM (5 μM, Cat# ab142780, Abcam), a Ca^2+^ indicator, and were incubated at 37°C for 30 min. After washing, the monolayers were loaded with RH 237 (15 μM, Cat# S1109, ThermoFisher), a potentiometric dye, and were incubated for an additional 30 min. The imaging solution contained (in mmol/L): 154 NaCl, 5.6 KCl, 2 CaCl_2_, 1 MgCl_2_, 8 D-glucose, and 10 HEPES, pH 7.3. An epifluorescence macroscope, equipped with two CMOS N256 cameras (MiCAM03, Brainvision, SciMedia Ltd., United States), was employed for the optical mapping system to capture action potentials (AP) and calcium transients (CaT) at a rate of 500 frames per second. The imaging setup included a 530 nm green LED light source (LEX2-LZ4-G), as well as an imaging cube comprising a collimator, a 560 nm dichroic mirror, and a 50 mm bandpass excitation filter from BrightLine^®^ by Semrock. To split the beam, a second imaging cube was employed, housing a 662-nm dichroic mirror, two emission filters (572/15 nm, BrightLine^®^, Semrock), a longpass filter (715 nm, Andover Corporation), and a lens system featuring a maximum aperture of f/1.4 for RH 237 and Rhod-2 imaging.

The monolayer was maintained at 37°C using a controlled heating plate (Multichannel Systems, Reutlingen, Germany) and was electrically stimulated using two platinum/iridium electrodes positioned at the lower edge of the monolayer. An STG4002 stimulus generator (Multichannel Systems) was used to deliver 10-ms square bipolar pulses, with an amplitude of 8 V. Initially, the spontaneous electrical activity was recorded, and then 10 µM blebbistatin (Cat# 331-20207-4, Cedarlane Labs) was added. The monolayers were then paced at 1.0 and 2.0 Hz. The raw optical signals were processed and were analyzed using Brainvision Workbench software (Brainvision, SciMedia). Activation times of each pixel were extracted for one AP/CaT propagation, which corresponds to the time at maximal depolarization rate and half-upstroke time, respectively. These extracted activation times were then utilized to create isochronal activation maps and to assess both conduction velocity (CV) and CaT propagation velocity (CaPV). In brief, two points, marking the start and end of the propagating wave (AP/CaT), were selected. Measurements were taken of the distance between these points and the disparity in activation times. CV was calculated by dividing the distance by the time difference. APDs and TDs were measured in each pixel as the duration between activation and 50% or 80% repolarization/reuptake times. Spontaneous initiation of sustained spiral waves was observed in the DM1 monolayers.

### 2.8 Statistical analysis

PRISM10 software (GraphPad, CA, United States) was used for all statistical analyses. The normality of the distribution was assessed using the D'Agostino-Pearson normality test. The data are expressed as medians ± quartiles (25% and 75%) with min to max values. The number of differentiations used for the different experiments was mentioned as replicate (*N*). For the qPCR and Western blot data, a replicate (*n*) corresponded to an RNA or protein extract. For the patch-clamp data, the number of recorded cells corresponded to a replicate (*n*). For the optical mapping data, a replicate (*n*) corresponded to a hiPSC-CM monolayer in a 24-well plate.

For two independent variables, such as the normalized intensity/voltage relationships (I/V), a two-way ANOVA with a Šídák multiple comparisons test was used (Figures 3B, 4B). Otherwise, a one-way ANOVA with a Tukey’s multiple comparisons test was used to compare the three groups. When the replicate (*n*) was too small (qPCR and Western blot data), a non-parametric Kruskal-Wallis test with a Dunn’s multiple comparisons test was performed to compare the three groups. Qualitative variables, such as the percentage of cells exhibiting events or spiral waves (Figure 8), were analyzed using a chi-square test. All statistical tests were conducted with a 95% confidence interval, and differences were considered significant beyond the 0.05% risk threshold (*p < 0.05, **p < 0.01, ***p < 0.001, and ****p < 0.0001).

## 3 Results

### 3.1 Patient description

The two patients with DM1 received a clinical diagnosis in adulthood. They exhibited common symptoms such as abnormal electromyography results, myotonia, muscle weakness, early cataracts, and cardiac electrical disorders. The first patient, a 55-year-old male (CBRCULi002-A or DM1-1290), experienced mild distal muscle weakness along with cervicalgia, brachialgia, weakness in the neck dorsiflexors, and heart conduction abnormalities. The DM1-1290 patient-specific iPSCs contained 1291 CTG repeats in the expanded allele of the *DMPK*. The second patient, a 30-year-old male (CBRCULi004-A or DM1-1640), exhibited proximal muscle weakness and a first-degree AV block. The DM1-1640 iPSCs displayed 1639 CTG repeats in the expanded allele of the *DMPK*. To serve as a control for this group of patients, we used CBRCULi001-A (CTRL), derived from an apparently healthy 44-year-old male. As pointed above, we recently expanded and characterized the hiPSC lines (Jauvin et al., 2023). For the cardiac differentiation of hiPSCs, we employed a reliable and reproducible commercial cardiac differentiation kit (StemCell). To enhance atrial specification in hiPSC-cardiomyocytes (hiPSC-CMs), we directly introduced RA between D2 and D5, leveraging its known ability (Devalla et al., 2015).

### 3.2 Molecular phenotypic hallmarks observed in DM1 hiPSC-CMs

**FIGURE 1:**
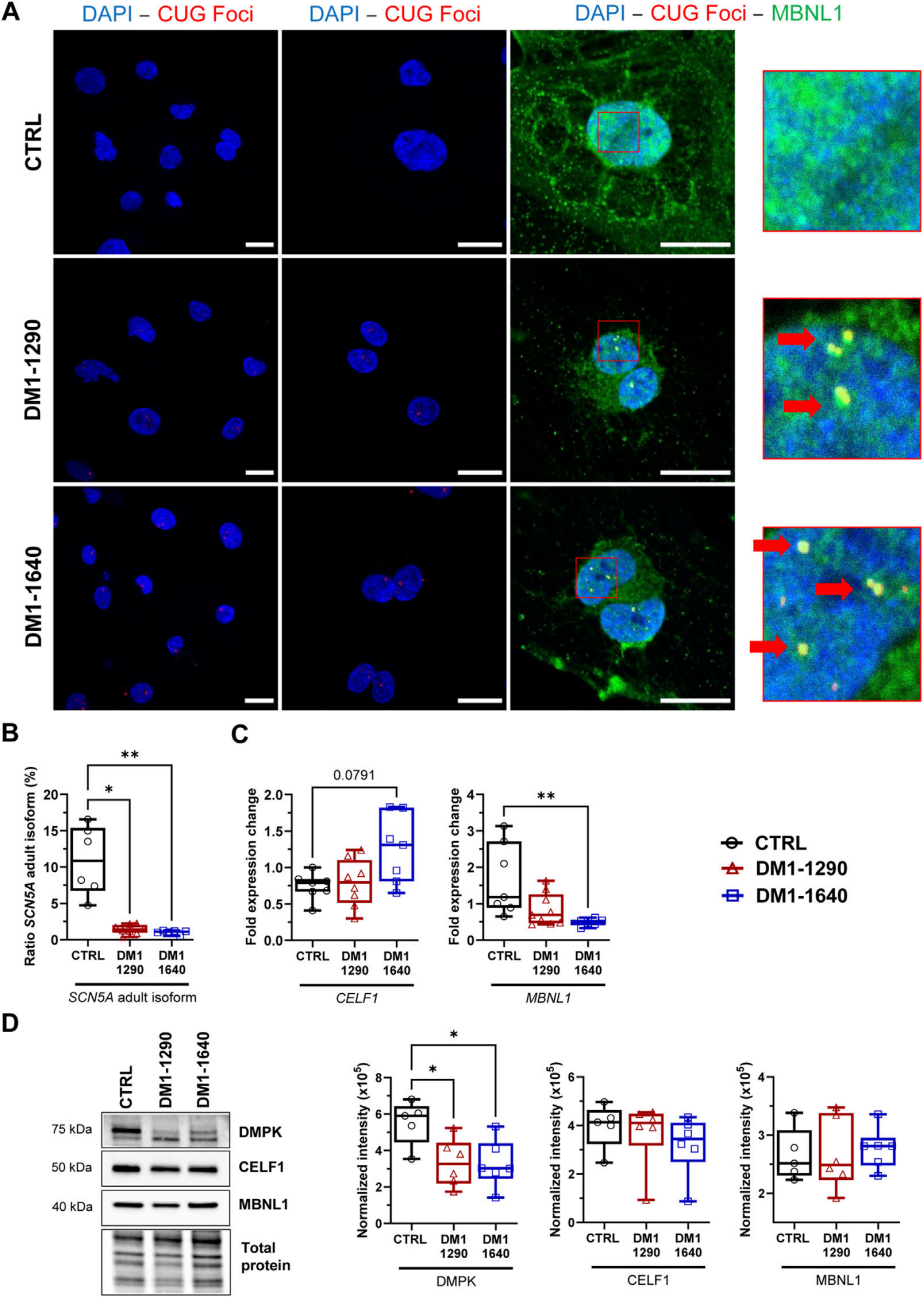
DM1 molecular hallmarks in DM1 hiPSC-CMs. **(A)** Dual fluorescence *in situ* hybridization (FISH) and immunostaining of CUG foci (red) and MBNL1 protein (green) in the nuclei (blue, DAPI) of DM1 hiPSC-CMs. Scale bar: 20 µm. Left panel: ×40; middle panel: ×63; right panel: ×100. Insets show magnifications of nuclei that reveal the colocalization of CUG foci and MBNL1 (with red arrows). **(B)** Percentage of the *SCN5A* mRNA adult isoform calculated as the ratio of exon 6b (adult isoform) to exon 6a (fetal isoform) multiplied by 100 and determined using qPCR measurements. CTRL (*N* = 5, *n* = 6), DM1-1290 (*N* = 6, *n* = 8), and DM1-1640 hiPSC-CMs (*N* = 5, *n* = 7). **(C)** qPCR analysis of the genes *MBNL1* and *CELF1* encoding the RNA-binding proteins MBNL1 and CELF1 in CTRL (*N* = 5, *n* = 7), DM1-1290 (*N* = 6, *n* = 8), and DM1-1640 (*N* = 5, *n* = 7) hiPSC-CMs. **(D)** Western blot analysis of DMPK, MBNL1, and CELF1 expression. Box and whiskers showing the quantification of proteins. Each protein is normalized to the total protein of its lane. CTRL (*N* = 5), DM1-1290 (*N* = 6), and DM1-1640 hiPSC-CMs (*N* = 5, *n* = 6). *p < 0.05 and **p < 0.01 as determined using a non-parametric Kruskal-Wallis test with Dunn’s multiple comparisons test. The replicate (*N*) corresponds to the number of differentiations while the replicate (*n*) corresponds to an RNA or protein extract.

Nuclear foci accumulation and their colocalization with MBNL1 are key pathological features of DM1. To assess the formation of nuclear foci, dual immunofluorescence *in situ* hybridization (FISH) staining was performed on DM1 hiPSC-CMs using MBNL1 and TNNT2 as cardiac markers (Figure 1A, Supplementary Figure S2). The analysis revealed the presence of foci formed by CUG-expanded RNAs in the nuclei of DM1-1290 and DM1-1640 hiPSC-CMs, while no foci were detected in the nuclei of control (CTRL) cells (Figure 1A). MBNL1 was observed in the nucleus and cytoplasm of CTRL hiPSC-CMs, whereas MBNL1 punctate staining (indicated by red arrows) was colocalized with nuclear CUG foci in DM1-1290 and DM1-1640 hiPSC-CMs (Figure 1A). This colocalization highlighted MBNL1 sequestration by the CUG-expended RNAs in the DM1 cell lines. To investigate the impact of CUG-RNA-induced toxicity on the transcriptome, we analyzed the splicing profile of *SCN5A*, which has previously been reported as mis-spliced in DM1 patients (Wahbi et al., 2013; Freyermuth et al., 2016). RT-qPCR was performed using specific primers targeting the fetal (exon 6a) and adult (exon 6b) isoforms of *SCN5A* mRNA. The CTRL hiPSC-CMs expressed 10.9% of the adult isoform, whereas DM1-1290 and DM1-1640 cells expressed 1.4% and 1.0% of the adult isoform, respectively (Figure 1B). The downregulation of the adult isoform in the two DM1 cell lines implies that the fetal isoform was upregulated. The mRNA and protein levels of MBNL1 and CELF1 were determined. No significant change was observed for CELF1 expression (Figures 1C, D). *MBNL1* mRNA level was reduced 3.5-fold in the DM1-1640 hiPSC-CMs (Figure 1C), but no noticeable difference was observed at protein levels (Figure 1D). Furthermore, the expression of the DMPK protein was also analyzed (Figure 1D). DMPK protein expression was 1.6-fold and 1.7-fold lower in the DM1-1290 and DM1-1640 hiPSC-CMs, respectively, compared to the CTRL hiPSC-CMs (Figure 1D).

### 3.3 Alteration of atrial and ventricular action potential properties in DM1 hiPSC-CMs

**FIGURE 2:**
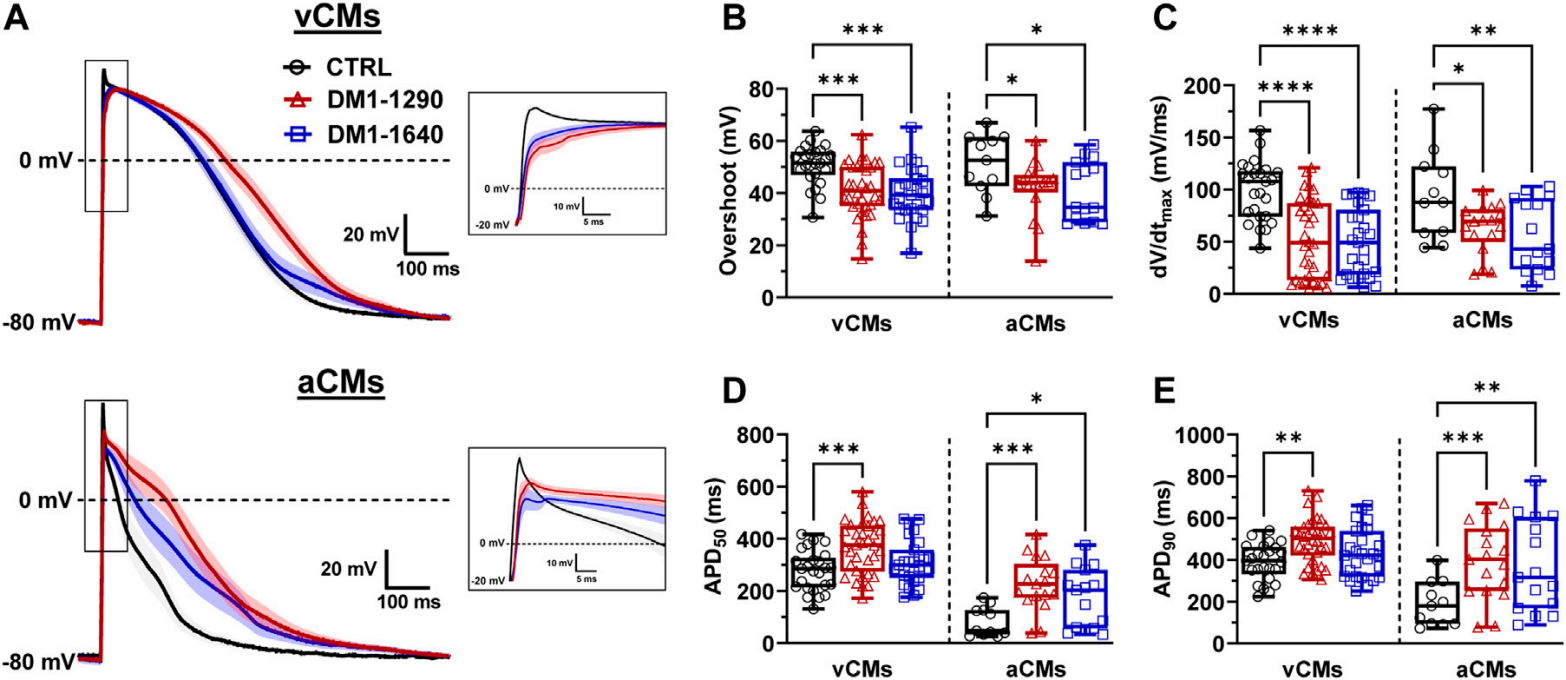
Alteration of atrial and ventricular action potential properties in DM1 hiPSC-CMs. **(A)** Superposed APs recorded in current-clamp mode at 1.0 Hz in ventricular (vCMs) and atrial (aCMs) CTRL (*N* = 5, 3, *n* = 25, 11), DM1-1290 (*N* = 6, 3, *n* = 34, 17), and DM1-1640 (*N* = 5, 3, *n* = 27, 15). Traces represent the mean ± SEM. Insets show a close-up view of APs. The holding potential was maintained at −80 mV. **(B–E)** Box and whiskers summarizing the overshoot **(B)**, the maximal upstroke velocity (dV/dt_max_) **(C)**, and the APD at 50% **(D)** and 90% of repolarization **(E)**. *p < 0.05, **p < 0.01, ***p < 0.001, and ****p < 0.0001 as determined using a one-way ANOVA with Tukey’s multiple comparisons test. The replicate (*N*) corresponds to the number of differentiations while the replicate (*n*) corresponds to the recorded cells for vCMs and aCMs, respectively.

The properties of the APs were studied in vCMs and aCMs from the DM1 cell lines which exhibited spontaneous APs. Comparing the resting membrane potential (RMP) between DM1-1290 and CTRL, similar values were observed in both vCMs and aCMs (Supplementary Table S2). However, in the case of DM1-1640 vCMs, a more depolarized RMP (−45.1 ± 2.1 mV) was recorded compared to CTRL vCMs (−52.1 ± 1.8 mV) (Supplementary Table S2). Conversely, no significant differences were found in aCMs RMP between DM1-1640 (−37.5 ± 4.1 mV) and CTRL (−34.5 ± 2.8 mV) (Supplementary Table S2). The following experiments were carried out on cardiomyocytes maintained at −80 mV and stimulated at 1.0 Hz to produce adequate APs with significant amplitudes and to ensure the suppression of spontaneous activity (Figure 2). APs were triggered in DM1-1290 and DM1-1640 hiPSC-CMs similar to CTRL hiPSC-CMs. However, the DM1 cell lines exhibited a higher threshold of depolarization compared to the CTRL cell line (Supplementary Table S2). Several AP parameters were analyzed, including overshoot, maximal upstroke velocity dV/dt_max_ (representing the opening of sodium channels, the phase 0 of APs), and 20%, 50%, and 90% of repolarization (corresponding to the opening of calcium and potassium channels, the 1st, 2nd, and 3rd phases of APs). The amplitude and the time to peak of the APs shown in the representative traces in Figure 2A were affected in vCMs and aCMs from DM1-1290 and DM1-1640 hiPSC-CMs. Specifically, the overshoot was decreased by 27% in vCMs and by 19% in aCMs (Figure 2B, Supplementary Table S2). DM1-1290 and DM1-1640 exhibited a significant decrease in dV/dt_max_ (DM1-1290 vCMs: 53.4 ± 6.4 mV/ms; aCMs: 62.9 ± 5.7 mV/ms; DM1-1640 vCMs: 49.6 ± 6.2 mV/ms; aCMs: 56.0 ± 8.8 mV/ms) compared to the CTRL (vCMs: 99.1 ± 5.6 mV/ms; aCMs: 94.2 ± 12.6 mV/ms) (Figure 2C, Supplementary Table S2). There was a marked increase in AP duration (APD) at 20%, 50%, and 90% of repolarization in both the vCMs and the aCMs in DM1-1290. However, in DM1-1640, the increase in APDs was only observed in the aCMs (Figures 2D, E, Supplementary Table S2). These outcomes were obtained during 1.0 Hz stimulation. Nevertheless, current-clamp recordings were also performed at stimulation frequencies of 1.5 and 2.0 Hz. It was observed that, when subjected to stimulation frequencies exceeding 1.0 Hz, the ability of DM1 hiPSC-CMs to synchronize with the stimulation rhythm was frequently compromised. Specifically, at 1.5 Hz stimulation frequency, the rhythm adaptation was hindered in 38% of DM1-1290 and 39% of DM1-1640 cases, in contrast to the 0% observed in CTRL hiPSC-CMs. Subsequently, at 2.0 Hz stimulation frequency, an even more pronounced effect was evident, with 72% of DM1-1290 and 79% of DM1-1640 failing to adapt to the stimulation rhythm, in comparison to the mere 3% observed in CTRL hiPSC-CMs. These findings show that the parameters of APs in the vCMs and the aCMs were altered in both DM1-1290 and DM1-1640.

### 3.4 Decreased sodium current density in DM1 hiPSC-CMs

**FIGURE 3:**
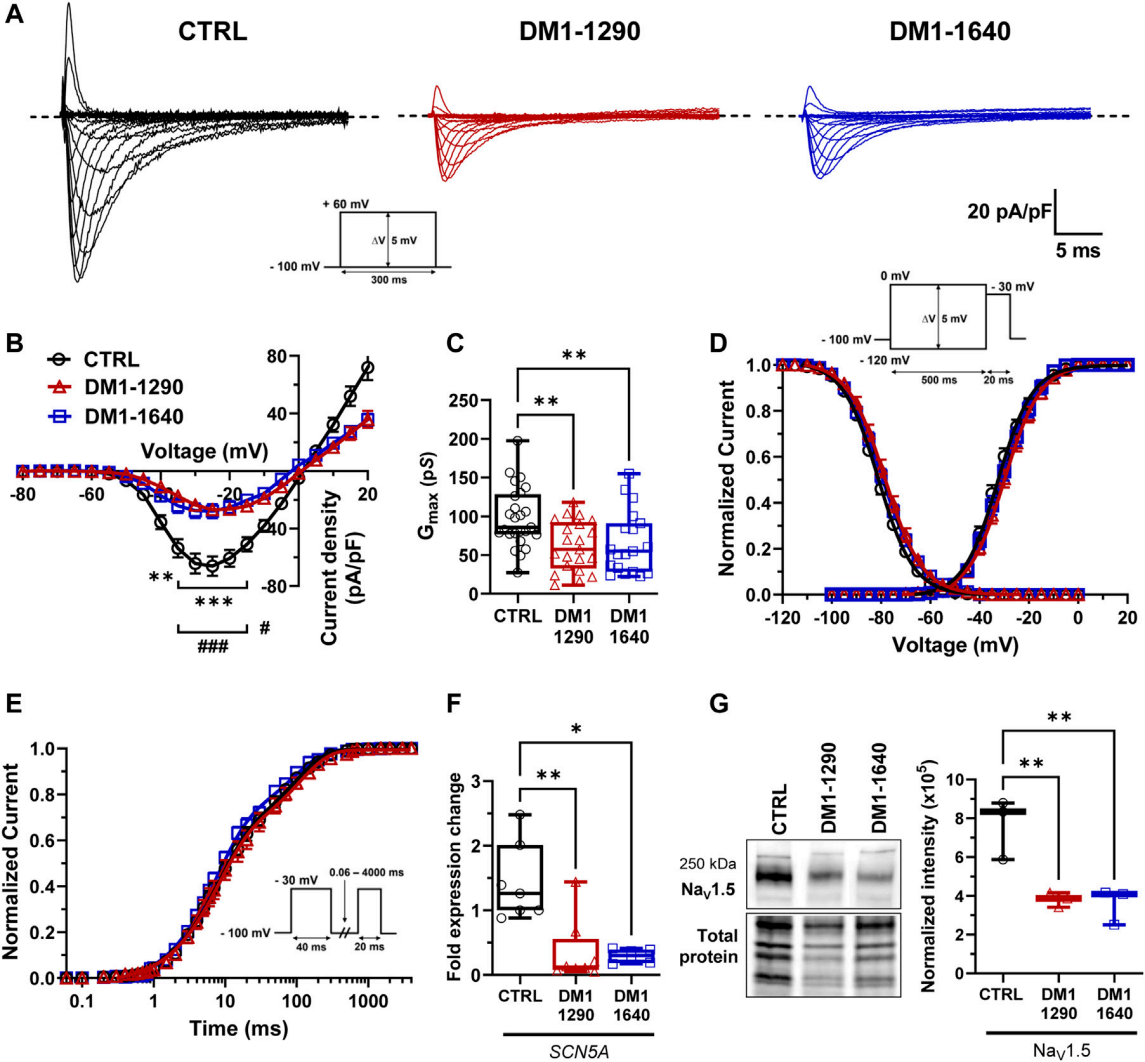
Decrease in Na_V_1.5 channel density in DM1 hiPSC-CMs. **(A)** Representative sodium currents (I_Na_). The dashed line represents zero current. The currents were obtained using 300-ms pulses from −100 mV to +60 mV in 5-mV increments. **(B)** Normalized intensity (I_Na_)/voltage relationships (I/V) in CTRL (*N* = 5, *n* = 25), DM1-1290 (*N* = 6, *n* = 21), and DM1-1640 (*N* = 5, *n* = 19) hiPSC-CMs. Sodium current densities were measured by normalizing current amplitudes to membrane capacitance. **p < 0.01, ***p < 0.001 (CTRL vs. DM1-1290), and #*p* < 0.05, ###< 0.001 (CTRL vs. DM1-1640). The significance was determined using a two-way ANOVA with Šídák’s multiple comparisons test. **(C)** Conductance of sodium currents. **p < 0.01 as determined using a one-way ANOVA with Tukey’s multiple comparisons test. **(D)** Steady-state activation and inactivation of sodium currents. Inactivation currents were obtained using 20-ms test pulses to −30 mV after a 500-ms pre-pulse to potentials ranging from −120 mV to 0 mV. **(E)** Recovery from inactivation. The cells were depolarized to −30 mV for 40 ms from a holding potential of −100 mV to inactivate the Na_V_1.5 channels. Test pulses were then applied at −30 mV for 20 ms to measure current amplitudes, with an interval ranging from 0.06 to 4000 ms. **(F)** qPCR analysis of *SCN5A* mRNA expression in CTRL (*N* = 5, *n* = 7), DM1-1290 (*N* = 6, *n* = 8), and DM1-1640 (*N* = 5, *n* = 6) hiPSC-CMs. *p < 0.05, and **p < 0.01 as determined using a non-parametric Kruskal-Wallis test with Dunn’s multiple comparisons test. **(G)** Western blot analysis of Na_V_1.5 channel expression. Box and whiskers showing Na_V_1.5 channel expression normalized to total protein for CTRL (*N* = 3), DM1-1290 (*N* = 3), and DM1-1640 hiPSC-CMs (*N* = 3). **p < 0.01 as determined using a non-parametric Kruskal-Wallis test with Dunn’s multiple comparisons test. The replicate (*N*) corresponds to the number of differentiations while the replicate (*n*) corresponds to either the number of recorded cells or RNA or protein extract.

The main contribution to the initiation and triggering of APs in cardiomyocytes has been attributed to Na_V_1.5 channels. As the *SCN5A* gene, which encodes Na_V_1.5 channels, was mis-spliced in DM1 hiPSC-CMs (Figure 1B), we further explored the biophysical properties of Na_V_1.5 by voltage-clamp mode experiments (Figure 3). Interestingly, the DM1-1290 and DM1-1640 vCMs produced lower I_Na_ currents than the CTRL (Figure 3A). The I_Na_ current density-voltage (I/V) relationship revealed a 2.4-fold decrease in both DM1-1290 and DM1-1640 hiPSC-CMs compared to CTRL hiPSC-CMs (Figure 3B, Supplementary Table S3). Additionally, the conductance was 1.5-fold lower in the DM1 cell lines (CTRL: 98.4 ± 7.8 pS; DM1-1290: 61.5 ± 7.0 pS; DM1-1640: 65.5 ± 9.2 pS) (Figure 3C, Supplementary Table S3). However, no significant difference was observed in the gating properties of the three groups (Figures 3D, E, Supplementary Table S3). The RT-qPCR results show that *SCN5A* mRNA expression was 4.2-fold and 4.9-fold lower in the DM1-1290 and DM1-1640 hiPSC-CMs, respectively, compared to the CTRL hiPSC-CMs (Figure 3F). The downregulation of *SCN5A* was confirmed by Western blot, where a significant decrease in Na_V_1.5 channel levels was observed in the DM1 cell lines (Figure 3G). These results show that there was a decrease in the Na_V_1.5 channel expression in the DM1 cell lines, which was consistent with the observed decrease in I_Na_ density at the single-cell level.

### 3.5 Alteration of voltage-gated calcium channel gating properties in DM1 hiPSC-CMs

**FIGURE 4:**
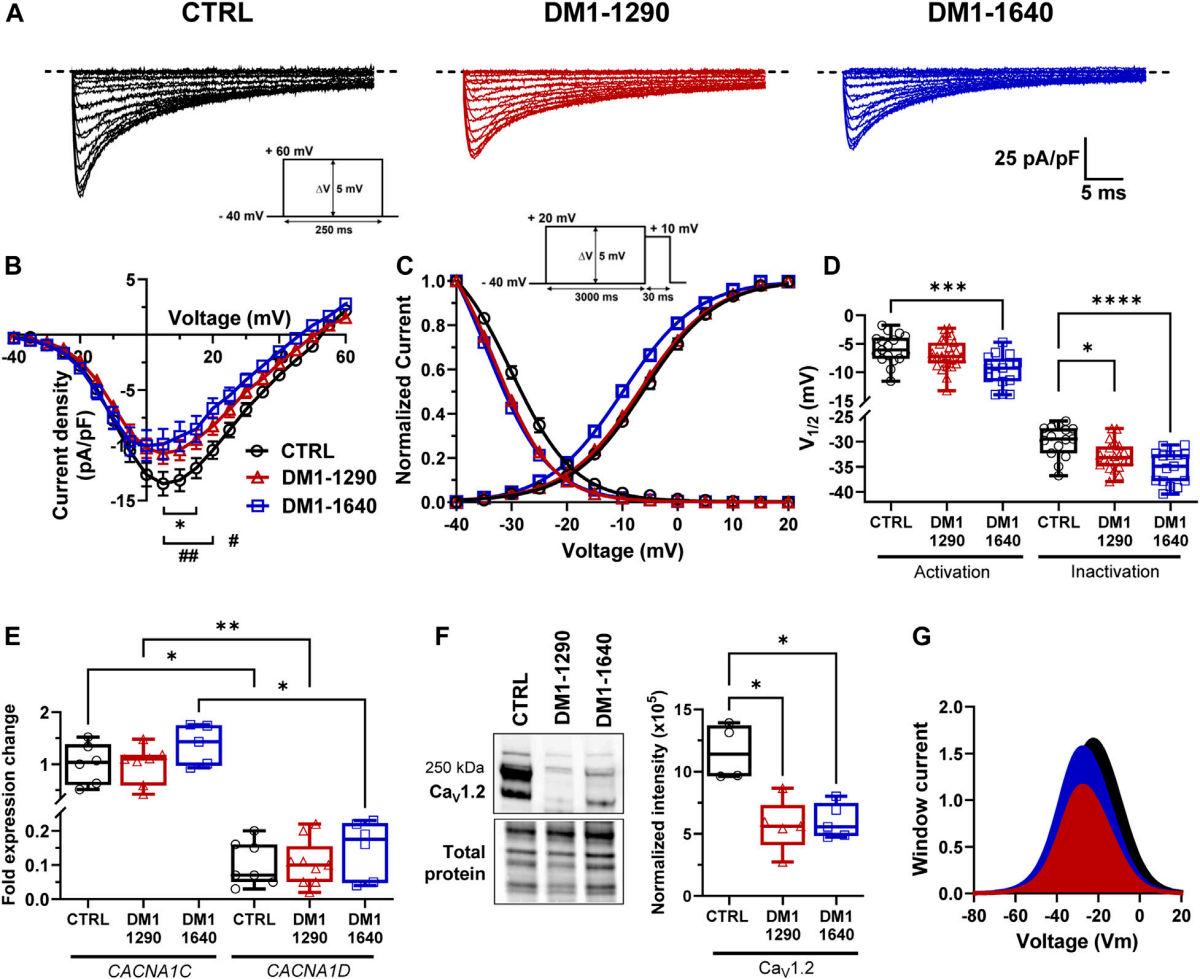
Alteration of voltage-gated calcium channel gating properties in DM1 hiPSC-CMs. **(A)** Representative calcium currents. The dashed line represents zero current. The currents were obtained using 250-ms pulses from −40 mV to +60 mV in 5-mV increments. **(B)** Normalized intensity (I_CaL_)/voltage relationships (I/V) in CTRL (*N* = 4, *n* = 16), DM1-1290 (*N* = 5, *n* = 29), and DM1-1640 (*N* = 4, *n* = 17) hiPSC-CMs. Calcium current densities were measured by normalizing current amplitudes to membrane capacitance. *p < 0.05 (CTRL vs. DM1-1290), and #*p* < 0.05, ##< 0.01 (CTRL vs. DM1-1640). The significance was determined using a two-way ANOVA with Šídák’s multiple comparisons test. **(C)** Steady state of activation and inactivation of I_CaL_ currents. Inactivation currents were obtained using 30-ms test pulses to +10 mV after a 3,000-ms pre-pulse to potentials ranging from −40 mV to +20 mV. **(D)** Box and whiskers summarizing the half-activation and half-inactivation potentials. *p < 0.05, **p < 0.01, and ***p < 0.001 as determined using a one-way ANOVA with Tukey’s multiple comparisons test. **(E)** qPCR analysis of *CACNA1C* and *CACNA1D* mRNA expression. *CACNA1D* is normalized to *CACNA1C* expression in CTRL (*N* = 5, *n* = 7), DM1-1290 (*N* = 6, *n* = 9), and DM1-1640 (*N* = 5, *n* = 6) hiPSC-CMs. *p < 0.05, and **p < 0.01 as determined using a non-parametric Kruskal-Wallis test with Dunn’s multiple comparisons test. **(F)** Western blot analysis of Ca_V_1.2 channel expression. Box and whiskers showing the quantification of Ca_V_1.2 channel expression normalized to the total protein for CTRL (*N* = 4), DM1-1290 (*N* = 5), and DM1-1640 hiPSC-CMs (*N* = 5). *p < 0.05 as determined using a non-parametric Kruskal-Wallis test with Dunn’s multiple comparisons test. **(G)** Window current representing the overlap between the activation and inactivation curves. The replicate (*N*) corresponds to the number of differentiations while the replicate (*n*) corresponds to either the number of recorded cells or RNA or protein extract.

The voltage-gated L-type calcium channels (VGCCs) play a crucial role in excitation-contraction coupling in cardiac muscle. The inward calcium current (I_CaL_) triggers the activation of Ryanodine receptors, which are responsible for the release of calcium from the sarcoplasmic reticulum, ultimately governing muscle contraction. We characterized the L-type calcium channels and their biophysical properties in the DM1 vCMs (Figure 4). A decrease in I_CaL_ density occurred in both DM1-1290 (−10.3 ± 0.9 pA/pF) and DM1-1640 (−9.3 ± 1.3 pA/pF) compared to the CTRL vCMs (−14.0 ± 1.0 pA/pF) (Figures 4A, B, Supplementary Table S4). However, there was no difference in conductance (Supplementary Table S4). The analysis of steady-state activation revealed a significant 3.8-mV shift toward hyperpolarized voltages in DM1-1640 but not in DM1-1290 (Figures 4C, D, Supplementary Table S4). The inactivation of I_CaL_ was affected in the DM1 cell lines, as indicated by a significant shift of the steady-state inactivation curves by 2.3 and 5.8 mV to more hyperpolarized voltages in the DM1-1290 and DM1-1640 hiPSC-CMs, respectively (Figures 4C, D, Supplementary Table S4). mRNA expression of the two main L-type calcium channels in the heart, *CACNA1C* and *CACNA1D*, which encode the Ca_V_1.2 and Ca_V_1.3 channels, respectively, was measured. The mRNA expression of both genes was similar across all three groups, but the overall *CACNA1D* mRNA expression was significantly lower than *CACNA1C* mRNA (Figure 4E). A decrease in Ca_V_1.2 channel expression was observed in the two DM1 cell lines by Western blot (Figure 4F). Indeed, the expression was 2-fold and 1.9-fold lower in DM1-1290 and DM1-1640 compared to CTRL hiPSC-CMs, respectively. These findings demonstrate that there was a decrease in Ca_V_1.2 channel expression in the DM1 cell lines, which was consistent with the smaller recorded I_CaL_ density associated with an alteration in gating properties. These alterations led to decrease in window current, leading to a loss-of-function for the VGCCs, particularly in DM1-1290 hiPSC-CMs (Figure 4G).

### 3.6 Sarcomeric organization and disruption of gap junction expression in DM1 hiPSC-CMs

**FIGURE 5:**
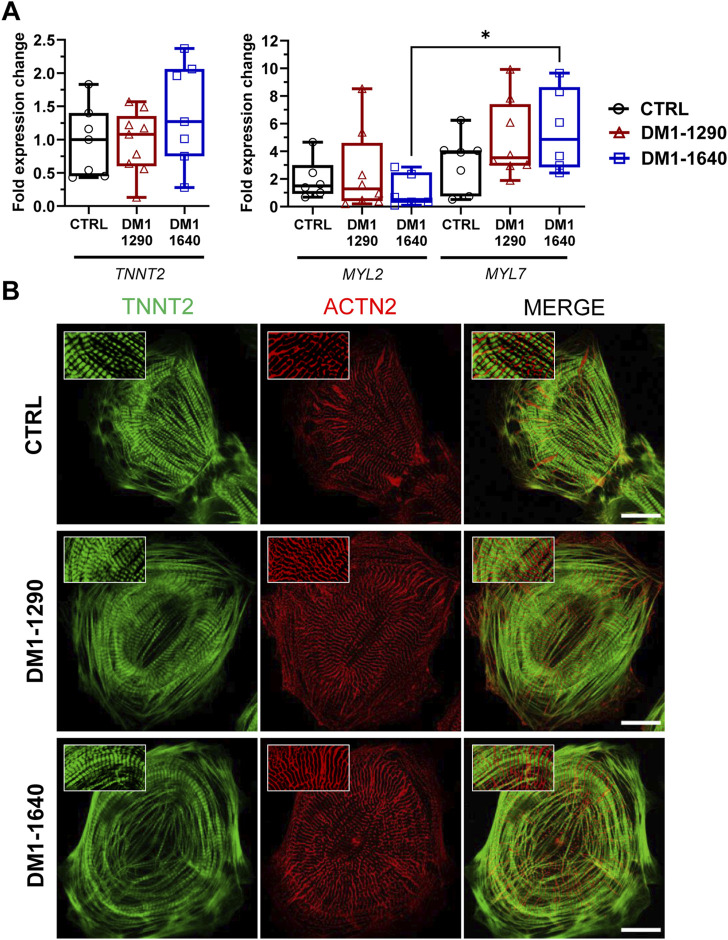
Intact contractile structures in DM1 hiPSC-CMs. **(A)** qPCR analysis of cardiac gene expression (*TNNT2, MYL2, MYL7*). *MYL7* is normalized to *MYL2* expression in CTRL (*N* = 5, *n* = 7), DM1-1290 (*N* = 6, *n* = 8), and DM1-1640 (*N* = 5, *n* = 6) hiPSC-CMs. *p < 0.05 as determined using a non-parametric Kruskal-Wallis test with Dunn’s multiple comparisons test. The replicate (*N*) corresponds to the number of differentiations while the replicate (*n*) corresponds to an RNA extract. **(B)** Immunofluorescence staining of cardiac markers ACTN2 (red) and TNNT2 (green). The last column shows merged images. The insets show a zoom-in of the stained proteins. Scale bar: 20 μm.

**FIGURE 6:**
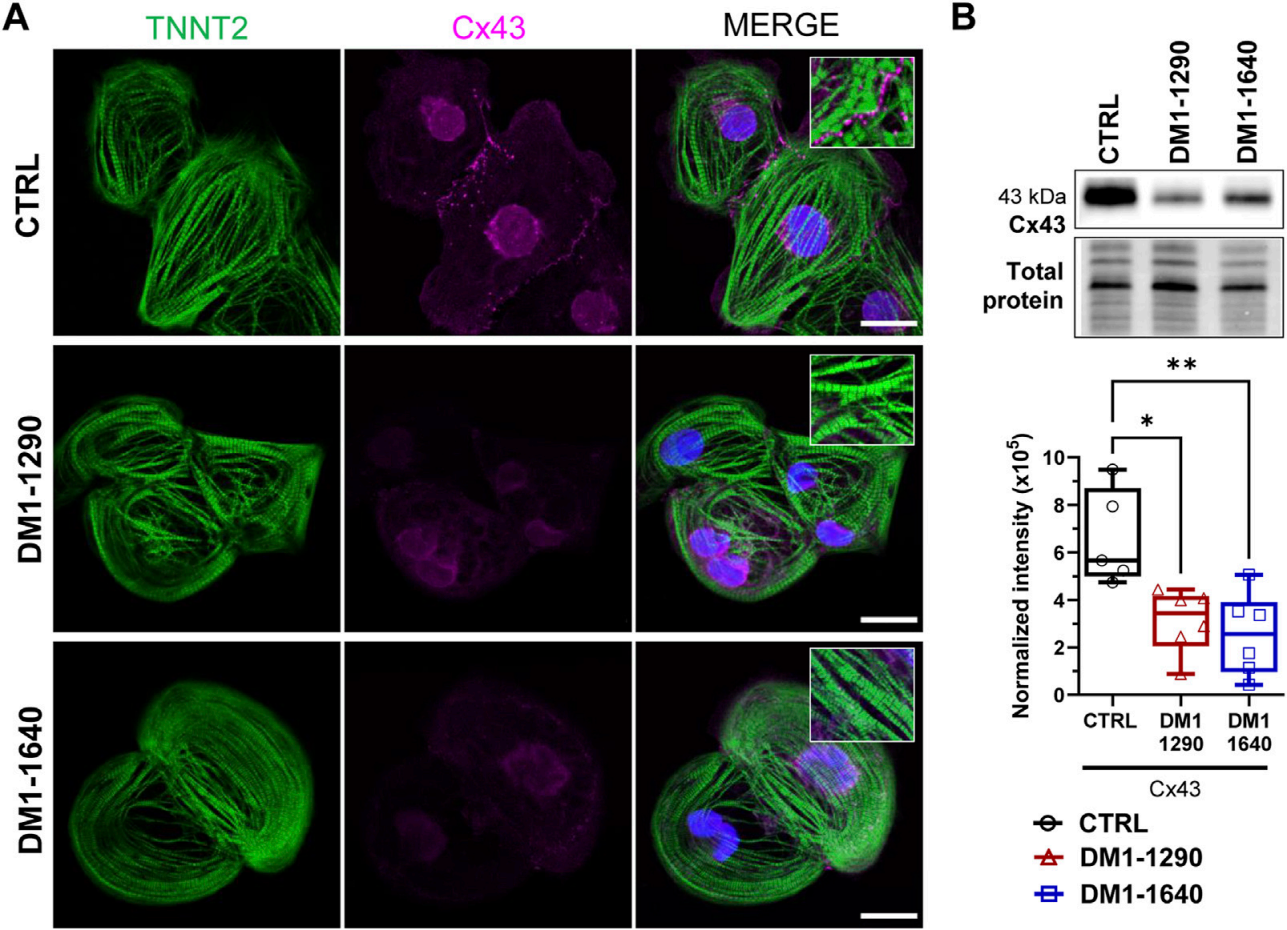
Disruption of gap junction proteins on the membrane of DM1 hiPSC-CMs. **(A)** Immunofluorescence staining of GJA1 protein (also known as Cx43, magenta) and TNNT2 (green). DAPI staining of the cell nuclei (blue). The last column shows merged images. The insets show a zoom-in of a punctate pattern of Cx43 located at the plasma membrane in the CTRL hiPSC-CM that was not detected in the DM1 hiPSC-CMs. Scale bar: 20 μm. **(B)** Western blot analysis of Cx43 expression. Box and whiskers showing the quantification of Cx43 expression normalized to the total protein for CTRL (*N* = 5), DM1-1290 (*N* = 6), and DM1-1640 hiPSC-CMs (*N* = 5, *n* = 6). *p < 0.05, and **p < 0.01 as determined using a one-way ANOVA with Tukey’s multiple comparisons test. The replicate (*N*) corresponds to the number of differentiations while the replicate (*n*) corresponds to a protein extract.

We next evaluated the contractile and sarcomeric organization of vCMs (Figure 5). mRNA expression of contractile genes (*TNNT2, MYL2, MYL7*) was assessed by RT-qPCR (Figure 5A). No changes were observed with *TNNT2*, *MYL2*, and *MYL7* mRNA expression in the DM1 cell lines, compared to the CTRL hiPSC-CMs. However, *MYL7* mRNA levels were significantly higher than *MYL2* mRNA levels in DM1-1640 hiPSC-CMs (Figure 5A). Sarcomeric organization was evaluated by immunofluorescence staining of ACTN2 and TNNT2 (Figure 5B). Morphologically, the immunostaining for the ACTN2 and TNNT2 proteins suggests that the CTRL and DM1 hiPSC-CMs had normal sarcomeric organizations. We next evaluated the gap junction alpha 1 protein expression (GJA1), also known as connexin 43 (Cx43), which is part of the gap junction family. The immunostaining of Cx43 revealed a perinuclear and a punctate pattern at the plasma membrane in CTRL hiPSC-CMs that were not detected in the DM1 hiPSC-CMs (Figure 6A). The loss of Cx43 expression was confirmed by Western blot, where a significant decrease in Cx43 protein levels was observed in DM1 hiPSC-CM (Figure 6B). Cx43 expression was 2-fold and 2.6-fold lower in the DM1-1290 and DM1-1640 hiPSC-CMs than in the CTRL hiPSC-CMs, respectively.

### 3.7 Slowed conduction and impaired calcium-transient propagation in DM1 monolayers

**FIGURE 7:**
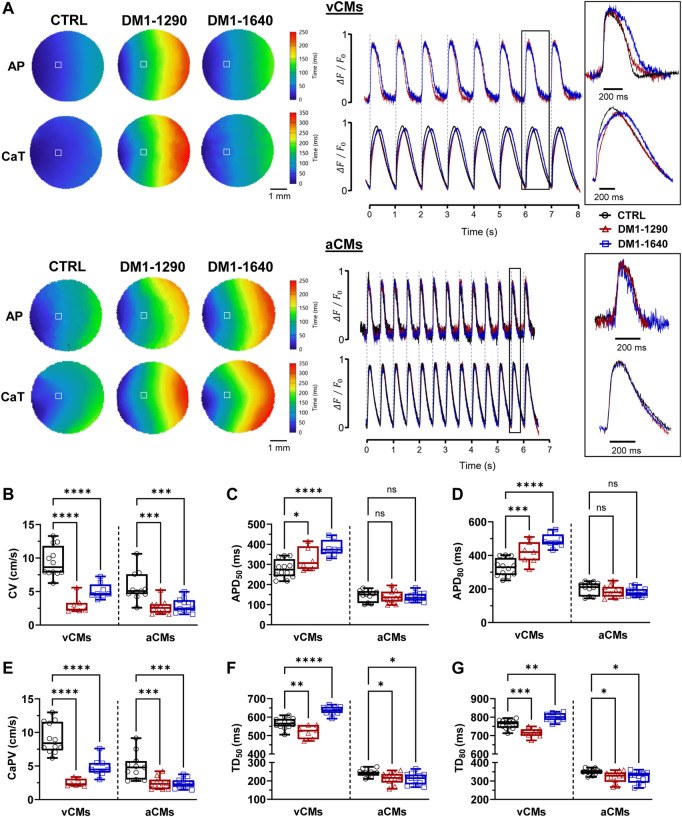
Slowed conduction, impaired CaT propagation, and altered AP and CaT parameters in DM1 monolayers. **(A)** Left panel: representative activation maps of APs and CaT propagation. Right panel: representative APs and CaT traces. vCM monolayers were paced at 1.0 Hz, and aCM monolayers were paced at 2.0 Hz. The insets show a close-up view of one AP and calcium transient. The symbol □ showing the position (3 × 3 pixels) from where the representative traces were recorded. **(B–D)** Box and whiskers summarizing the conduction velocities **(B)** and the AP durations at 50% **(C)** and 80% **(D)** of repolarization. **(E–G)** Box and whiskers summarizing the calcium-transient propagation velocities **(E)** and the calcium-transient durations at 50% **(F)** and 80% **(G)** of the reuptake phase. CTRL (*N* = 3, 3, *n* = 12, 11), DM1-1290 (*N* = 3, 3, *n* = 7, 10), and DM1-1640 (*N* = 3, 3, *n* = 8, 11). *Ns* (no significant), *p < 0.05, **p < 0.01, ***p < 0.01 as determined using a non-parametric Kruskal-Wallis test with Dunn’s multiple comparisons test. The replicate (*N*) corresponds to the number of differentiations while the replicate (*n*) corresponds to an hiPSC-CM monolayer in a 24-well plate for vCMs and aCMs, respectively.

Cardiac conduction velocity is mainly regulated by Cx43 and Na_V_1.5 channels. Reduced Cx43 and Na_V_1.5 expressions can impair gap junction coupling, reduce excitability, and decrease the myocardial conduction velocity, which can lead to heart conduction disorders (Jansen et al., 2012). The electrophysiological abnormalities of DM1 hiPSC-CMs were assessed at the tissue level by generating ventricular and atrial monolayers. Simultaneous recordings of the APs and calcium-transient (CaT) propagation were taken using two different probes on the same monolayer using optical mapping technique. Prior to the addition of blebbistatin and electrical pacing, the spontaneous beating frequencies were measured for each monolayer. The beating frequencies were approximately 0.5 and 1.0 Hz in the three groups for the vCM and aCM monolayers, respectively (Supplementary Table S5). The activation maps as well as the representative APs and CaT traces (Figure 7A) showed uniform propagation throughout the entire vCM and aCM monolayers from the DM1 and CTRL hiPSC-CMs. Conduction velocities were significantly slower in the DM1 monolayers than in the CTRL monolayers for both aCM and vCM (Figure 7B, Supplementary Table S5). It should be emphasized that greater differences in CVs could potentially be observed in more mature hiPSC-CMs. The AP parameters showed that only DM1 vCM monolayers had prolonged APDs. APD_50_ and APD_80_ were significantly prolonged in the DM1-1290 and DM1-1640 monolayers (Figures 7C, D, Supplementary Table S5). CaT propagation velocities were also reduced in DM1 vCM and aCM monolayer compared to CTRL monolayer (Figure 7E, Supplementary Table S6). In addition, the CaT parameters of the DM1 monolayer were altered. In the vCM monolayers, DM1-1290 had a shortened CaT while DM1-1640 had a prolonged CaT (Figures 7F, G, Supplementary Table S6). The CaT durations at 50% (TD_50_) and 80% (TD_80_) of reuptake in the aCM monolayer were reduced in the DM1-1290 and DM1-1640 monolayer compared to the CTRL monolayer (Figures 7F, G, Supplementary Table S6). These findings show that DM1 monolayer had slower conduction velocities, altered AP parameters, and impaired CaT propagation in both vCMs and aCMs.

### 3.8 Arrhythmogenic events and spiral waves in DM1 hiPSC-CMs

**FIGURE 8:**
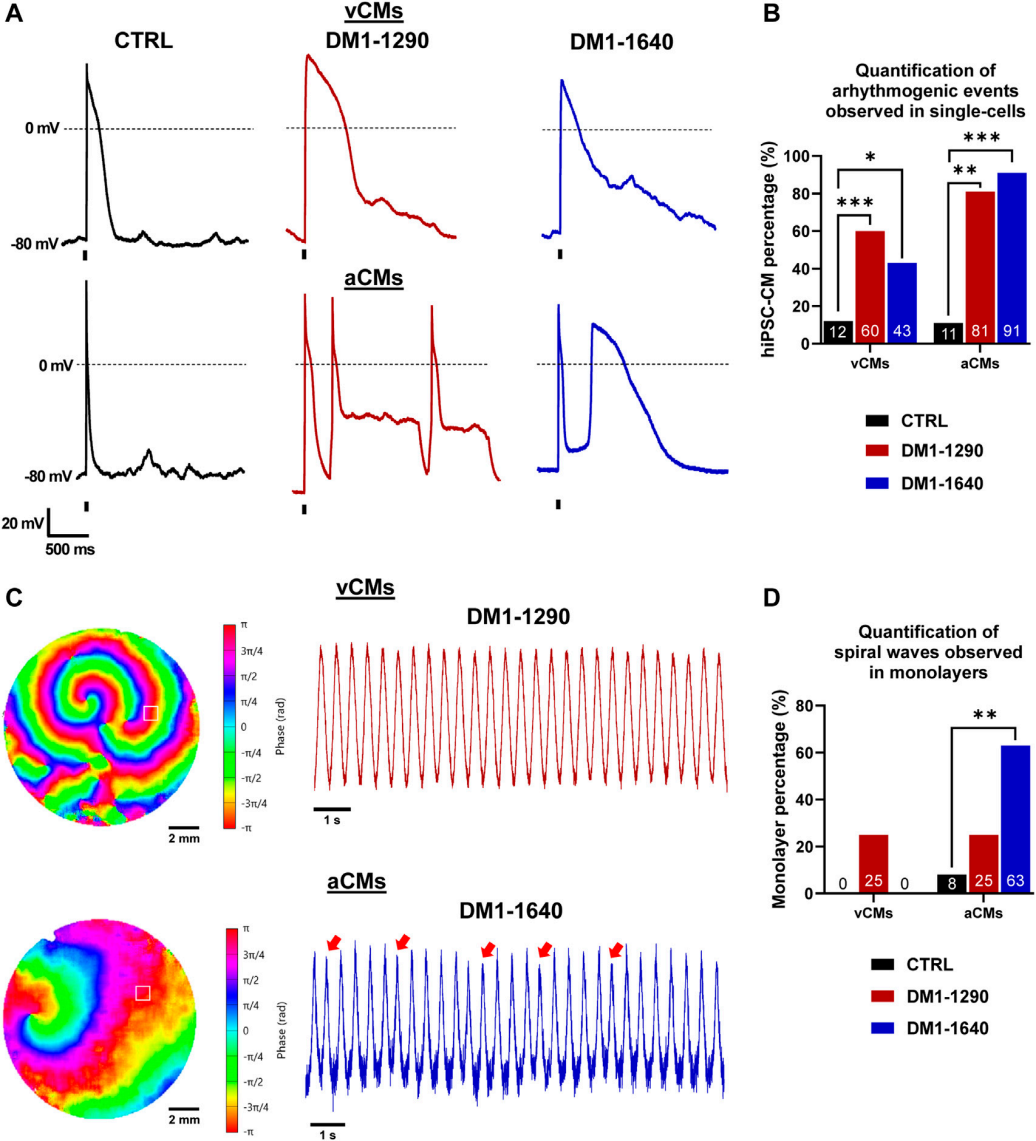
Arrhythmogenic events and spiral waves observed in DM1 hiPSC-CMs and monolayers. **(A)** Representative AP traces at a 0.5-Hz stimulation frequency exhibiting arrhythmogenic events. The holding potential was maintained at −80 mV. The vertical bars at the bottom of the traces indicate the 3-ms stimulation pulse. **(B)** Bar graph showing the percentage of cells exhibiting arrhythmogenic events. The number in the bottom bar represents the percentage for CTRL (*N* = 5, 3, *n* = 24, 22), DM1-1290 (*N* = 6, 3, *n* = 30, 19), and DM1-1640 (*N* = 5, 3, *n* = 23, 20). *p < 0.05, **p < 0.01, ***p < 0.01 as determined using a chi-square test. **(C)** AP phase maps and their traces showing the spontaneous initiation and sustaining of spiral waves. The red arrows show AP alternans with a smaller amplitude. The symbol □ showing the position (3 × 3 pixels) from where the representative traces were recorded. **(D)** Bar graph showing the percentage of monolayers exhibiting spiral waves. The number in the bottom bar represents the percentage for CTRL (*N* = 3, 3, *n* = 12, 12), DM1-1290 (*N* = 3, 3, *n* = 12, 12), and DM1-1640 (*N* = 3, 3, *n* = 9, 11). **p < 0.01 as determined using a chi-square test. The replicate (N) corresponds to the number of differentiations while the replicate (n) corresponds to either the number of recorded cells or hiPSC-CM monolayer in a 24-well plate for vCMs and aCMs, respectively.

The mechanisms underlying cardiac arrhythmias in DM1 hiPSC-CMs were investigated in a subsequent analysis. At the single-cell level, DM1 hiPSC-CMs exhibited inherent instability and displayed spontaneous abnormal events in both vCMs and aCMs (Supplementary Figure S3A). The hiPSC-CMs were paced at 0.5 Hz to quantify the proportion of cells exhibiting arrhythmogenic events. DM1 hiPSC-CMs exhibited early-afterdepolarizations (EADs) and delayed-afterdepolarizations (DADs), while only a few DADs were noticed in some CTRL hiPSC-CMs (Figure 8A, Supplementary Figure S3B). Specifically, arrhythmogenic events in vCMs were triggered in 60% of DM1-1290 and 43% of DM1-1640 hiPSC-CMs whereas the proportion increased in aCMs, with 81% of DM1-1290 and 91% of DM1-1640 hiPSC-CMs exhibiting such events (Figure 8B). At the tissue level, the initiation of spontaneous sustained spiral waves was only observed in the DM1 monolayers (Figure 8C, Supplementary Videos S1, S2). The AP phase maps clearly illustrate the disorganized electrical activity that occurred in the DM1 monolayer characterized by multiple, slowly moving, splitting, and colliding rotors. AP recordings of DM1-1640 aCMs displayed AP alternans with a reduced amplitude (red arrows, Figure 8C). The percentage of monolayer exhibiting spiral waves was then quantified. In vCMs, 25% of DM1-1290 monolayer triggered spiral waves, which was not statistically significant (Figure 8D). However, in aCMs, 25% of the DM1-1290 and 63% of the DM1-1640 monolayers exhibited spiral waves. The data indicate that DM1 monolayers exhibited disorganized electrical activity. These findings were more pronounced in DM1-1640, suggesting that this cell line has a larger degree of atrial electrical abnormalities.

## 4 Discussion

We investigated the cardiac involvement of two new myotonic dystrophy type 1 (DM1) patient-specific induced pluripotent stem cell lines (hiPSCs). We examined the molecular hallmarks of DM1, highlighting the accumulation of foci and the sequestration of MBNL1 in both DM1 hiPSC-CMs. We also observed mis-splicing of *SCN5A*, and separately characterized its impact on atrial and ventricular electrical activity, conduction, and calcium homeostasis. The two DM1 cell lines exhibited reduced sodium and calcium current densities, prolonged action potential duration, slower conduction velocity, and impaired calcium transient propagation in both vCMs and aCMs. Remarkably, arrhythmogenic events were recorded, and ventricular and atrial arrhythmias were observed in the two DM1 cell lines.

### 4.1 The impact of *SCN5A* mis-splicing on the properties of voltage-gated sodium channels

DM1 is caused by mutant CUG repeats in the *DMPK* gene resulting in an RNA gain-of-function and altered splicing regulation (Furling et al., 2003). Nuclear RNA foci containing expanded CUG repeats are present in DM1 human cardiac muscle and mouse models (Mankodi et al., 2005; Mahadevan et al., 2006; Rao et al., 2021). The findings show that nuclear foci accumulated in DM1 hiPSC-CMs and that DMPK protein expression was reduced. These foci colocalized with MBNL1 protein, suggesting a loss-of-function of this RNA-binding protein. Surprisingly, we did not observe any significant differences in CELF1 protein expression between the CTRL and the DM1 hiPSC-CMs despite its upregulation in DM1 tissues (Timchenko et al., 2001). CELF1 is upregulated in the heart of DM1 mouse models, and its overexpression replicates numerous functional and molecular DM1 defects (Wang et al., 2007; Koshelev et al., 2010). However, the relative immaturity of the hiPSC-CMs may account for the absence of CELF1 overexpression in the DM1 cell lines compared to the CTRL cell lines, as CELF protein expression is known to be elevated during embryogenesis and fetal heart development (Kalsotra et al., 2008).

At the molecular level, RNA toxicity in DM1 is primarily associated with numerous gene mis-splicing that result in the overexpression of embryonic isoforms in adult tissues and cardiomyocyte cultures (Mankodi et al., 2005; Freyermuth et al., 2016; Kim et al., 2019; Wang et al., 2019). One of the prominent splicing defects involves *SCN5A*, the gene encoding the Na_V_1.5 channel. Dominant mutations in *SCN5A*, such as missense, non-sense, splice site, and deletion/duplication mutations, lead either to a gain or a loss of function and cause various cardiac conduction abnormalities (Li et al., 2018). These conduction abnormalities share similarities with those observed in DM1 individuals. In DM1, the switch from the adult to the fetal isoform of *SCN5A* transcripts results in an increased proportion of fetal *SCN5A* transcripts (Wahbi et al., 2013; Freyermuth et al., 2016; Poulin et al., 2021). MBNL proteins directly regulate the alternative splicing of *SCN5A* (Dixon et al., 2015; Freyermuth et al., 2016). Despite the immaturity of the hiPSC-CMs, we confirmed that splicing switches the adult isoform to the fetal isoform in *SCN5A* mRNAs in DM1 hiPSC-CMs compared to CTRL hiPSC-CMs. However, unlike the DM1-1300 hiPSC-CMs (Poulin et al., 2021) in our previous study, we did not observe any changes in the gating properties of Na_V_1.5 channels caused by the increased expression of the fetal isoform. We instead found a shift of activation to depolarized voltages, similar to the shift observed between neonatal and adult isoforms (Onkal et al., 2008; Freyermuth et al., 2016). However, both DM1-1290 and DM1-1640 exhibited a 60% decrease in I_Na_ density, which was likely caused by a lower Na_V_1.5 channel density at the cell surface. Similar reductions in I_Na_ density have been observed in *Xenopus* oocytes, EBNA-293 cells, and hiPSC-CMs when the adult *SCN5A* isoform is switched to the fetal isoform (Onkal et al., 2008; Freyermuth et al., 2016; Veerman et al., 2017). Differences in protein incorporation between fetal and adult *SCN5A* isoforms could potentially arise from distinct membrane targeting signals or varying post-translational modifications, both of which influence Na_V_1.5 channel trafficking. It is worth noting that the fetal isoform of our CTRL hiPSC-CMs amounted to 89.1% of the baseline level but increased to 98.6% and 99% in DM1-1290 and DM1-1640 hiPSC-CMs, respectively. This high baseline level could potentially mask the changes seen in the gating properties that resulted from the switch from the adult to the fetal isoform in our cell lines. Enhancing the maturity of hiPSC-CMs in a 3D microtissue environment promotes postnatal *SCN5A* maturation (Campostrini et al., 2023), which offers a potential solution for addressing the limitations of the immature phenotype of hiPSC-CMs.

### 4.2 Alteration of atrial and ventricular action potential properties

Reduced I_Na_ currents significantly affect APs by altering their triggering and rising phase (phase 0). DM1 hiPSC-CMs displayed a slower rising phase (dV/dt_max_) and a reduced overshoot in vCMs. In aCMs, the overshoot was slightly reduced. Similar AP profiles were observed in DM1-1290 and DM1-1640. The AP of the DM1-1290 cell line was more altered than that of DM1-1300 (Poulin et al., 2021). The loss of excitability due to a Na_V_1.5 channel deficiency was confirmed by the fact that a stronger injected current was required to trigger APs from DM1 hiPSC-CMs. Indeed, Na_V_1.5 knock-out in hiPSC-CMs has been shown to raise the depolarization threshold (Pierre et al., 2021). The entry of sodium into cardiomyocytes triggers a series of ion channel activations, including potassium and calcium channels, which contribute to the plateau and repolarization phases of APs.

The significance of APD arises from its role in the initiation and perpetuation of arrhythmias, with its influence being paramount. Arrhythmogenic circuits are driven by variations in APD across diverse regions of the heart, laying the foundation for their formation. Prolonged APD, as in long QT syndrome, heightens the risk of life-threatening ventricular arrhythmias, while shortened APD, as in short QT syndrome, promotes premature beats and reentry (András et al., 2021). We found distinct outcomes according to the experiments carried out. In electrophysiological recordings, only DM1-1290 showed prolonged APD of vCMs whereas DM1 cell lines both exhibited this prolongation in optical mapping analysis. Additionally, a prolonged APD was exclusively observed at the single-cell level in DM1 aCMs. This dissimilarity could be explained by the heterogeneity of cardiac cell types at the monolayer level. In fact, the selection based on APD_90_ for CTRL revealed that 60% of vCMs displayed a ventricular-like AP shapes, 35% displayed atrial-like AP shapes, and 5% displayed nodal-like AP shapes. Treatment with RA shifted cell populations into 20% ventricular-like APs, 70% atrial-like APs and 10% nodal-like APs. Unlike our earlier research (Poulin et al., 2021), we observed an increase in the APD of vCMs for DM1-1290, which was possibly due to the decrease in Na_V_1.5 channel density observed in our study. We previously demonstrated that the complete absence of Na_V_1.5 in hiPSC-CMs leads to a longer APD in patch-clamp experiments (Pierre et al., 2021), which is consistent with reports of prolonged QTc intervals in DM1 patients (Park et al., 2013). In addition, the forced expression of the fetal isoform in adult mouse hearts induces a slower conduction velocity, an increase in APD, and prolonged PR and QRS intervals (Pang et al., 2018). The switch from the adult to the fetal isoform also leads to a decrease in I_Na_ and an increase in APD in hiPSC-CMs (Veerman et al., 2017). The generation of a persistent sodium current by the fetal *SCN5A* isoform has been demonstrated in cancer cells (Guzel et al., 2019). Nevertheless, no substantial persistent sodium current was detected in DM1 hiPSC-CMs (Data not shown). As a result, no association can be established between this current and the observed APD elevation in DM1 hiPSC-CMs. Finally, the increase in APD has also been observed in *Mbnl1* ^−/−^ and *Mbnl2* ^+/−^ mice (Chou et al., 2017), suggesting that a MBNL loss-of-function mediated by RNA toxicity in DM1 cardiomyocytes plays a contributing role.

We observed a 36% decrease in I_CaL_ density in both DM1-1290 and DM1-1640 hiPSC-CMs, which is expected to shorten the APD and slightly counter the prolongation due to the decrease in I_Na_ current density. However, our findings differ from previous observations as we detected an increase in Ca_V_1.2 at both the transcript and protein levels, which led to an increase in I_CaL_ density in DM1-1300 (Poulin et al., 2021). Similar increases in I_CaL_ have been reported in the skeletal and heart muscles of DM1 patients (Rau et al., 2011; Tang et al., 2012). A shift in the inactivation curve to depolarized voltages in DM1 hiPSC-CMs leading to a gain-of-function of Ca_V_1.2 has also been reported (Poulin et al., 2021). However, our outcomes show that the inactivation curve was shifted to hyperpolarized voltages and that the activation curve was shifted to depolarized voltages in both DM1 cell lines, indicating a loss-of-function of Ca_V_1.2. These findings agree with a previous study of hiPSC-CMs showing that *CACNA1C* is downregulated (Spitalieri et al., 2018). Taken together, this suggests that calcium channels can be either upregulated or downregulated in DM1 patients depending on the study model used and the patients from which iPSC-CMs were derived from, all of which may contribute to the molecular complexity of the disease. Therefore, our results and previous reports have shown that a heterogenous and complex dysregulation of calcium channel activity is involved in the pathogenesis of cardiac symptoms in DM1.

### 4.3 Abnormal conduction, disruption of gap junction proteins, and arrhythmogenic events

The two DM1 patients were known with cardiac conduction abnormalities. The 30-year-old male patient with 1640 CTG repeats had a first-degree AV block, which is present in 28.2%–34.1% of DM1 patients (Petri et al., 2014). However, the 55-year-old male patient with 1291 CTG repeats had unspecified conduction abnormalities. An AV block refers to abnormally slow conduction through the AV node characterized by a PR interval greater than 200 ms, with no disruption of atrial and ventricular conduction. To reproduce the conduction defects in DM1 patients, we conducted simultaneous voltage and calcium optical mapping in hiPSC-CM monolayers. Our results show that there was a significant decrease in CVs, which was correlated with a slower CaT propagation and was consistent with our previous findings (Poulin et al., 2021). The recorded CVs in CTRL hiPSC-CMs remain relatively slow, primarily attributed to their immaturity. More profound disparities in CVs could manifest in more fully developed hiPSC-CMs (Ahmed et al., 2020). Nevertheless, it is worth mentioning that hiPSC-CM monolayers have shown variable CVs across different hiPSC-CM lines, ranging from 2 to 20 cm/s, as reported in previous studies. (Shaheen et al., 2018; Li et al., 2020). Conduction defects are prevalent in DM1 and are associated with a high risk of sudden death. Studies on a DM1 mouse models have shown the presence of conduction defects (Mahadevan et al., 2006; Lee et al., 2013; Rao et al., 2021; Lee et al., 2022) and a slower conduction velocity (Chou et al., 2017). The decrease in CV and CaT propagation that we observed might be related to the reduced expression of Na_V_1.5 channels and gap junction proteins in our DM1 cell lines. We previously showed that the complete absence of Na_V_1.5 channels leads to conduction velocity slowing in hiPSC-CMs (Pierre et al., 2021). The primary gap junctions in the heart are GJA1 protein (Cx43), which is expressed throughout the myocardium, and GJA5 protein (Cx40), which is restricted to the atrial and cardiac conduction system (Lo, 2000). Deficiencies in connexins result in cardiac conduction defects. Our immunostaining and Western blot analyses revealed that there was a significant decrease in Cx43 expression in DM1 vCMs compared to CTRL vCMs. In addition, RT-qPCR showed that there was a decrease in *GJA5* mRNA levels in DM1 aCMs compared to CTRL aCMs (data not shown). Several studies on DM1 mice have also shown that Cx43 and Cx40 are downregulated in heart tissue (Kasahara et al., 2001; Yadava et al., 2008; Wang et al., 2009; Rao et al., 2021). The downregulation of connexins in DM1 may be attributed to various mechanisms. Overexpression of the cardiac transcription factor NKX2-5 induced by RNA toxicity may lead to progressive heart block and the downregulation of the connexins (Kasahara et al., 2001; Wakimoto et al., 2002; Yadava et al., 2008). Cx43 levels are partially restored in DM1 mice treated with a PKC inhibitor (Wang et al., 2009). The DMPK and Cx43 are colocalized at the intercalated disks in rat cardiac muscle (Schiavon et al., 2002), suggesting that DMPK haploinsufficiency may directly impact Cx43 expression at the membrane. It is well established that Cx43 colocalizes with Na_V_1.5 channels at the intercalated discs of cardiomyocytes, suggesting that there is a physical interaction between the two proteins (Agullo-Pascual et al., 2014). As such, the reduction in Na_V_1.5 channels may contribute to the alteration of the expression/distribution of Cx43, and impaired conduction in the heart. Furthermore, in another model of dilated cardiomyopathy, CELF1 was found to regulate *GJA1* mRNA degradation through an interaction with the nuclear-specific exoribonuclease RRP6, leading to Cx43 downregulation (Chang et al., 2017).

Reduced conduction velocity and prolonged APDs contribute to unidirectional block, leading to re-entrant arrhythmias. Abnormal repolarization in DM1 is associated with afterdepolarizations, which increase the risk of triggered arrhythmia and sudden cardiac death (Milner et al., 1991). Torsades de Pointes (TdP), a lethal ventricular arrhythmia, can be triggered by DADs, and especially EADs in patients with long QT syndrome (Weiss et al., 2010; Kurata et al., 2019). Our research on DM1 cell lines revealed disorganized electrical activity, with a high prevalence (43%–91%) of single-cell EADs and DADs. This is the first report of EADs in a DM1 hiPSC-CM model, which contrasts with a previous study that observed DADs in only 20% of DM1 ventricular hiPSC-CMs (Spitalieri et al., 2018). The authors used cells from DM1 patients without cardiac symptoms, whereas our patients exhibited conduction disorders. Additionally, we observed spiral waves in the aCMs and vCMs of DM1 monolayers. Spiral waves, also known as rotors, are complex wave patterns that can occur in the electrical activity of cardiac tissue. They form when an electrical signal propagates around a circuitous pathway, continuously re-entering and activating the same region of tissue (Pandit and Jalife, 2013). The presence of conduction block and re-entry has been demonstrated in a DM1 mouse model, which supports our findings with 2D monolayer of hiPSC-CMs (Chou et al., 2017). Furthermore, our results indicate that aCMs were more severely affected than ventricular vCMs in both DM1 cell lines, particularly in the cell line with 1600 CTG repeats. Both atrial and ventricular arrhythmias can occur with DM1. Atrial fibrillation, atrial flutter and atrial tachycardia are the most common, affecting approximately one-quarter of DM1 patients (Wahbi et al., 2016; Russo et al., 2021). Our differentiation method allows us to also investigate atrial specific electrical alterations in DM1 in sight to atrial involvement in these patients.

## 5 Conclusion

The present study provides important insights into the cardiac involvement of DM1 using new patient-specific hiPSCs, and highlights the crucial role of voltage-gated sodium channels in DM1 pathogenesis. Specifically, the overexpression of fetal isoforms of *SCN5A* transcripts results in decreased sodium current density, which alters the AP profiles of DM1 hiPSC-CMs. We observed significant alterations in atrial and ventricular AP properties such as slower rising phases, prolonged APDs, and reduced calcium currents. These modifications, along with the downregulation of gap junction proteins, may be related to the conduction defects and arrhythmogenic events witnessed in DM1 patients. The decrease in conduction velocities and the presence of EADs, DADs, and spiral waves in our DM1 hiPSC-CMs highlight the potential risk of ventricular and atrial arrhythmias associated with this disease.

Supported by prior investigations, a clear correlation can be established between the extent of mis-splicing occurrences, particularly to *SCN5A*, and the resultant conduction abnormalities within cardiomyocytes. It is conclusively affirmed that DM1 cell lines bearing 1300 CTG repeats or more exhibit substantial disturbances in voltage-gated ion channels, likely contributing to the cardiac manifestations observed in affected patients. Nevertheless, discrepancies persist in relation to the modulation of voltage-gated ion channels when compared with earlier studies, even among DM1 patients who exhibit similar CTG repeat lengths. This highlights the complexity of the disease and accentuates the need for an expansive inquiry encompassing a substantial cohort of DM1 patients. These findings offer essential understanding into the underlying pathophysiological mechanisms governing the cardiac implications in this disorder.

## 6 Study limitations

While the study effectively describes and validates the role of hiPSCs in investigating the pathological mechanisms underlying clinical aspects of DM1, it is important to note the limitations concerning the maturity of hiPSC-CMs. HiPSC-CMs still exhibit an immature or fetal phenotype. The electrophysiological characteristics of our cells (CTRL vCMs) align with previous studies and represent nearly mature human cardiomyocytes. Moreover, our study primarily aims to juxtapose the phenotypes of DM1 CMs with CTRL CMs; consequently, the paramount consideration in our viewpoint resides in the comparability among the hiPSC-CMs.

Utilizing the ventricular differentiation kit from STEMCELL Technologies offers reliable and reproducible cardiac differentiation. In atrial differentiation, the kit led to an overexpression of pacemaker-like CMs rather than atrial-like CMs (Darche et al., 2022). While various protocols incorporate retinoic acid for hiPSC differentiation into aCMs, our approach builds on a well-established protocol (Devalla et al., 2015), adding RA directly to the commercial protocol for its benefits. This modification allow us to attain a majority of atrial-like CMs; however, a certain degree of heterogeneity still persists at the monolayer level. This combination yields a standardized procedure, enhancing differentiation reproducibility across experiments and laboratories, yielding functional cardiomyocytes.

During single-cell AP recordings, a constant current maintained the membrane potential at −80 mV using the amplifier’s holding command mode. It is acknowledged that the application of a constant current to sustain a resting potential of −80 mV may not entirely replicate the effects of a K^+^ current and could potentially influence AP parameters. In this regard, the adoption of the dynamic clamp technique, characterized by real-time evaluation and injection of simulated membrane current, emerges as a potent tool in cardiac electrophysiology. Nonetheless, even without current injection in the AP measurements from optical mapping, we succeeded in achieving similar APD values between patch-clamp recordings and optical mapping recordings.

Another limitation is the absence of isogenic cells. The utilization of such methodologies could eliminate the impact of other genetic variations present in the patient cohort. However, our focus centered on investigating individuals who were either healthy or afflicted with DM1 from the Saguenay-Lac-Saint-Jean region (Quebec, Canada), where the prevalence of the condition is notably high (1:633) (Mathieu and Prévost, 2012).

## Data Availability

The original contributions presented in the study are included in the article/Supplementary Material, further inquiries can be directed to the corresponding author.
