# Supplementary material for: Cardiac involvement in patient-specific induced pluripotent stem cells of myotonic dystrophy type 1: unveiling the impact of voltage-gated sodium channels
Source: Front Physiol. 2023 Sep 18;14:1258318. doi: 10.3389/fphys.2023.1258318 (PMC10544896; doi:10.3389/fphys.2023.1258318)
Supplement: Supplementary file 3 [file DataSheet1.docx]

Supplementary Material

Cardiac involvement in patient-specific induced pluripotent stem cells of myotonic dystrophy type 1: unveiling the impact of voltage-gated sodium channels

**Marion Pierre^1^, Mohammed** **Djemai^1^, Charles-Albert Chapotte-Baldacci^1^, Valérie Pouliot^1^, Jack Puymirat^2^, Mohamed Boutjdir^3,4,5^, and Mohamed Chahine^1,6✉^**

*** Correspondence:** Mohamed Chahine, Ph.D. [Mohamed.Chahine@phc.ulaval.ca](mailto:Mohamed.Chahine@phc.ulaval.ca)

# Supplementary Data

## Legends for the video files

**Supplementary Video S1: Spiral waves in DM1-1290 vCMs.** Video of AP phase maps showing spontaneous initiation and sustenance of spiral waves.

**Supplementary Video S2: Spiral waves in DM1-1640 aCMs.** Video of AP phase maps showing spontaneous initiation and sustenance of spiral waves.

# Supplementary Figures and Tables

##
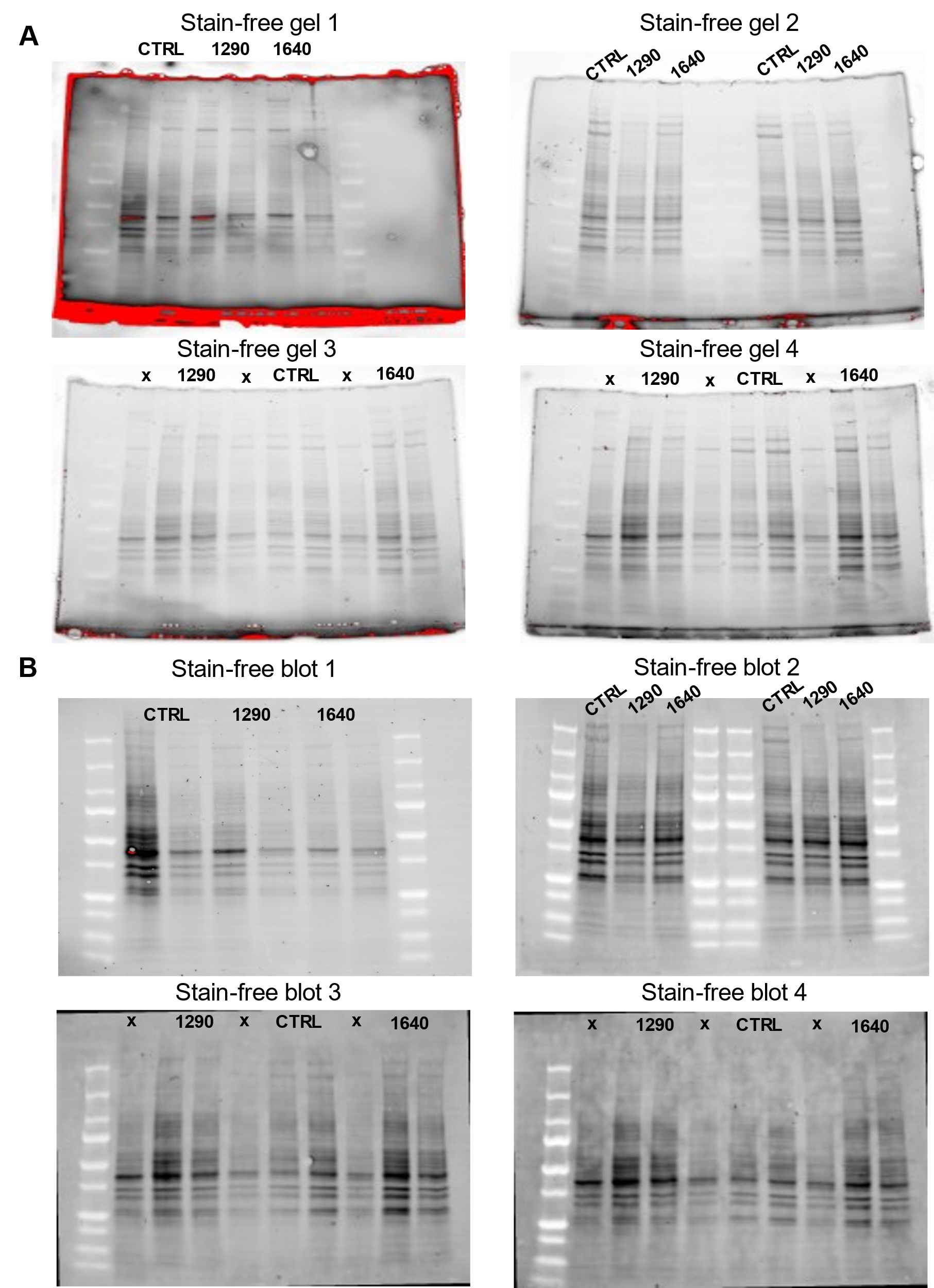
Supplementary Figures

**Supplementary Figure S1. Pictures of gels and blots used for the quantification of proteins.** **A:** Pictures of stain-free gels showing total proteins before transfer. **B:** Pictures of stain-free blots showing total proteins after protein transfer. The total protein was used to quantify the protein expression for each antibody. Protein ladder: Precision Plus Protein™ All Blue Prestained Protein Standards (Cat# 1610373, Bio-Rad). The conditions are written above. DM1-1290, DM1-1640, x: sample non-include.

**
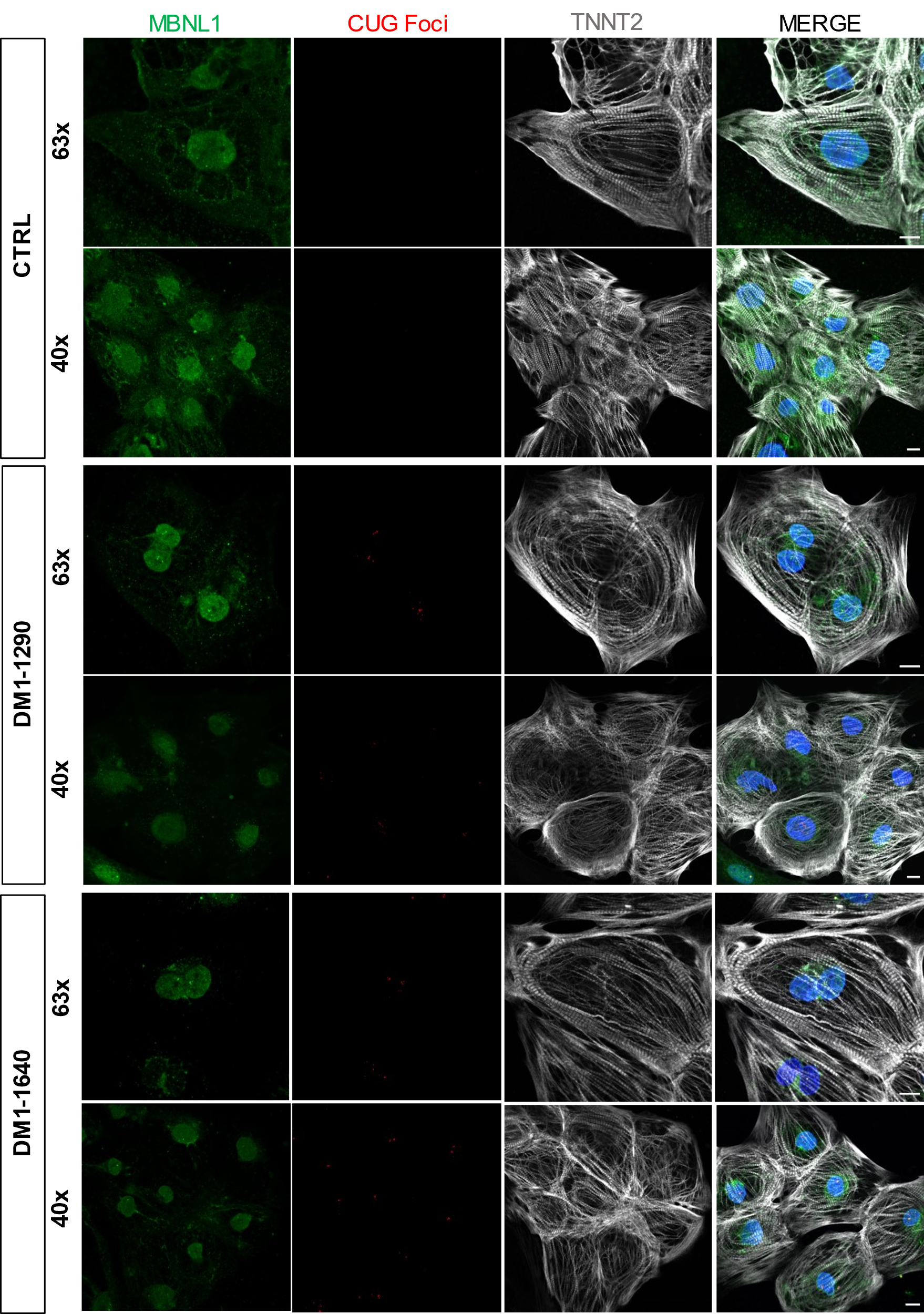
Supplementary Figure S2. Complete images of dual immunofluorescence (IF)-fluorescence *in situ* hybridization (FISH) staining shown in Figure 1.** TNNT2 (grey) staining was used to evaluate the presence of CUG foci/MBNL1 in the nuclei (blue) of cardiomyocytes. The last column shows merged images. Scale bar: 10 µm.


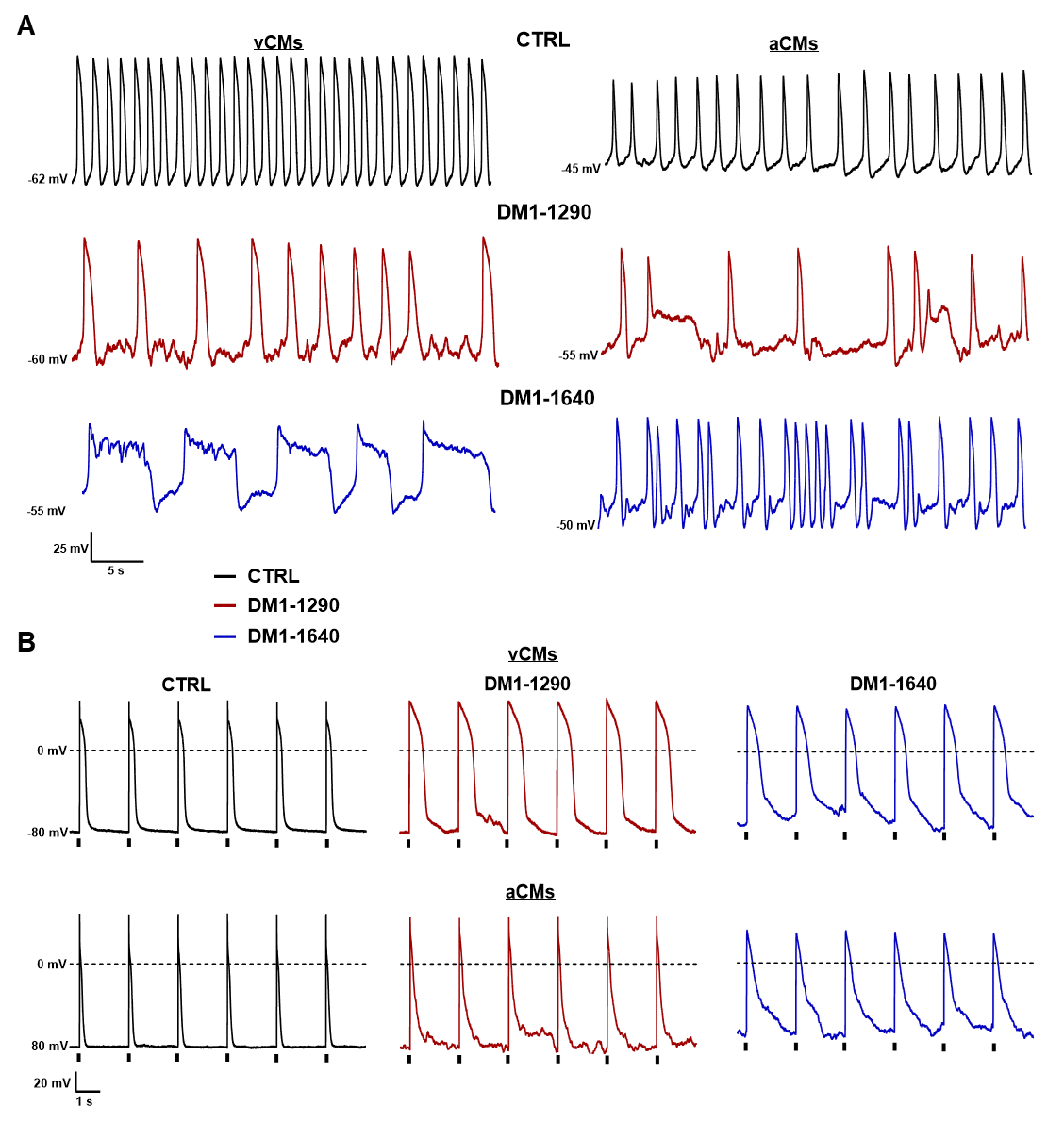
**Supplementary Figure S3. Arrhythmogenic events observed in spontaneous and stimulated current-clamp recordings from CTRL and DM1 hiPSC-CMs. A:** Representative spontaneous APs traces recorded in gap-free mode (I=0). The resting membrane potential (RMP) is indicated for each condition. **B:** Representative APs traces at 0.5-Hz stimulation frequency. The holding potential was maintained at -80 mV. Vertical bars at the bottom of the traces indicate the 3-ms stimulation pulse.

## Supplementary Tables

| **Supplementary Table S1. Human gene-specific primers for qPCR** | | | | |
| --- | --- | --- | --- | --- |
| **Gene** | **Primers (5' - 3')** | | **Tm (°C)** | **Size (bp)** |
|  | **Forward** | **Reverse** |  |  |
| *GATA4* | TCCCTCTTCCCTCCTCAAAT | TCAGCGTGTAAAGGCATCTG | 58 | 193 |
| *PPIA* | TTCATCTGCACTGCCAAGAC | TCGAGTTGTCCACAGTCAGC | 61 | 158 |
| *RPL22* | CCATGGCTCCTGTGAAAAA | TCACGGTGATCTTGCTCT | 61 | 219 |
| *CACNA1C* | GTCCAGCACACCTCCTTCAG | AGCCCCATAAGCAGTCATCTTC | 58 | 105 |
| *CACNA1D* | GCTGTTTGGCGGCAAGTTTA | CTTCGCCTGTCAGGATCTGG | 58 | 107 |
| *TNNT2* | AGAGGAGGAGGAGCTCGTTT | CCACTTTTCCGCTCTGTCTTC | 61 | 243 |
| *MYL2* | CCTTTCCACCATGGCACCT | AAGCCATCCCTGTTCTGGTC | 61 | 138 |
| *MYL7* | ATCTGCAAGGCAGACCTGAG | TGAAGTTGATGGGGCCCTTG | 61 | 109 |
| *CELF1* | GGATCAGACAAGTGCAGCAAG | GCCGTTCATTTTCTTTGAGACGG | 64 | 147 |
| *MBNL1* | CTGCCGAACATCTGACTAGC | TTGTGTGTGTTGCTTGACGA | 61 | 154 |
| *SCN5A* (exon 6a) | AGTATGTCGAGTACACCTTCAC | CTGAAAGTTCGAAGAGCCGAC | 61 | 193 |
| *SCN5A* (exon 6b) | GCATACACAACTGAATTTGTGG | GTCTTCAGCCCTGAAATGAC | 61 | 104 |
| *SCN5A* (exon 25) | ATGAAGAGCAGCCTCAGTGG | CCAATAAAGAGGTTCAGGGTGA | 58 | 100 |

Tm: melting temperature, bp: base pair, *GATA4*: GATA binding protein 4 *PPIA*: Peptidylprolyl Isomerase A, *RPL22*: Ribosomal Protein L22, *CACNA1C*: Calcium Voltage-Gated Channel Subunit Alpha1 C, *CACNA1D*: Calcium Voltage-Gated Channel Subunit Alpha1 D, *TNNT2*: Troponin T2, cardiac type, *MYL2*: Myosin Light Chain 2, *MYL7*: Myosin Light Chain 7, *CELF1:* CUG triplet repeat, RNA binding protein 1 (CUGBP) Elav-like family member 1*, MBNL1:* Muscleblind Like Splicing Regulator 1*, SCN5A*: Sodium Voltage-Gated Channel Alpha Subunit 5. Exon 6a: fetal isoform, exon 6b: adult isoform.

| Supplementary Table S2. Parameters of action potentials in vCMs and aCMs. | | | | | | | | |
| --- | --- | --- | --- | --- | --- | --- | --- | --- |
| Parameters | **Conditions** | | | | | | | |
|  | CTRL | | DM1-1290 | | | DM1-1640 | | |
|  | Mean  ± SEM | n | Mean  ± SEM | n | *p* value | Mean  ± SEM | n | *p* value |
| Action potentials (APs) in isolated vCMs | | | | | | | | |
| Membrane properties | | 25 | | 34 | | | 27 | |
| Resting membrane potential RMP (mV) | -52.1  ± 1.8 |  | -51.1  ± 2.1 |  | 0.9140  *ns* | -45.1  ± 2.1 |  | 0.0403  ∗ |
| Threshold of depolarization (nA) | 0.8  ± 0.04 |  | 1.0  ± 0.07 |  | 0.0370  ∗ | 0.8  ± 0.04 |  | 0.9832  *ns* |
| AP parameters | | 25 | | 34 | | | 27 | |
| Overshoot (mV) | 55.8  ± 1.5 |  | 41.2  ± 1.8 |  | 0.0008  ∗∗∗ | 39.8  ± 1.9 |  | 0.0003  ∗∗∗ |
| Max. upstroke vel. dV/dt_max_ (mV/ms) | 99.1  ± 5.6 |  | 53.4  ± 6.4 |  | <0.0001  ∗∗∗∗ | 49.6  ± 6.2 |  | <0.0001  ∗∗∗∗ |
| AP durations | | 25 | | 34 | | | 27 | |
| At 20% of repolarization APD_20_ (ms) | 131.1  ± 13.1 |  | 232.5  ± 12.6 |  | <0.0001  ∗∗∗∗ | 196.4  ± 12.3 |  | 0.0012  ∗∗ |
| At 50% of repolarization APD_50_ (ms) | 276.5  ± 15.6 |  | 368.8  ± 17.1 |  | 0.0005  ∗∗∗ | 310.1  ± 16.6 |  | 0.3479  *ns* |
| At 90% of repolarization APD_90_ (ms) | 389.5  ± 17.5 |  | 494.3  ± 18.2 |  | 0.0075  ∗∗ | 430.8  ± 22.6 |  | 0.4705  *ns* |
| Action potentials (APs) in isolated aCMs | | | | | | | | |
| Membrane properties | | 11 | | 17 | | | 15 | |
| Resting membrane potential RMP (mV) | -34.5  ± 2.8 |  | -37.5  ± 2.6 |  | 0.6716  *ns* | -37.5  ± 4.1 |  | 0.6716  *ns* |
| Threshold of depolarization (nA) | 0.2  ± 0.03 |  | 0.5  ± 0.05 |  | 0.0028  ∗∗ | 0.5  ± 0.03 |  | 0.0054  ∗∗ |
| AP parameters | | 11 | | 17 | | | 15 | |
| Overshoot (mV) | 51.4  ± 3.4 |  | 42.0  ± 2.6 |  | 0.0344  ∗ | 40.8  ± 3.0 |  | 0.0182  ∗ |
| Max. upstroke vel. dV/dt_max_ (mV/ms) | 94.2  ± 12.6 |  | 62.9  ± 5.7 |  | 0.0325  ∗ | 56.0  ± 8.8 |  | 0.0089  ∗∗ |
| AP durations | | 11 | | 17 | | | 15 | |
| At 20% of repolarization APD_20_ (ms) | 17.2  ± 5.7 |  | 110.8  ± 16.5 |  | 0.0008  ∗∗∗ | 91.0  ± 19.8 |  | 0.0123  ∗ |
| At 50% of repolarization APD_50_ (ms) | 83.0  ± 17 |  | 226.3  ± 24.7 |  | 0.0002  ∗∗∗ | 173.4  ± 29.4 |  | 0.0294  ∗ |
| At 90% of repolarization APD_90_ (ms) | 189.5  ± 32.2 |  | 392.7  ± 43.6 |  | 0.0003  ∗∗∗ | 370  ± 57.1 |  | 0.0019  ∗∗ |

Data are represented in mean ± SEM and the replicate (n) represents the number of recorded cells. The significance was determined using the one-way ANOVA with Tukey’s multiple comparisons test. The *p* value represents the adjusted *p* value from Tukey’s multiple comparisons test when the initial one-way ANOVA is significant. Otherwise, the *p* value come from the one-way ANOVA when it is no significant. *ns*: no significant, ∗*p*<0.05, ∗∗*p*<0.01, ∗∗∗*p*<0.001 and ∗∗∗∗*p*<0.0001. vCMs: ventricular-like cardiomyocytes derived from induced pluripotent stem cells, aCMs: atrial-like cardiomyocytes derived from induced pluripotent stem cells.

| Supplementary Table S3. Parameters of Na_V_1.5 channel biophysical properties. | | | | | | | | |
| --- | --- | --- | --- | --- | --- | --- | --- | --- |
| Parameters | **Conditions** | | | | | | | |
|  | CTRL | | DM1-1290 | | | DM1-1640 | | |
|  | Mean  ± SEM | n | Mean  ± SEM | n | *p* value | Mean  ± SEM | n | *p* value |
| Na_V_1.5 channel properties | | | | | | | | |
| I_Na_ currents | | 25 | | 21 | | | 19 | |
| Current density at -25 mV (pA/pF) | -66.1  ± 6.7 |  | -27.0  ± 3.8 |  | <0.0001  ∗∗∗∗ | -28.0  ± 4.9 |  | <0.0001  ∗∗∗∗ |
| Conductance G_max_ (pS) | 98.4  ± 7.8 |  | 61.5  ± 7.0 |  | 0.0028  ∗∗ | 65.5  ± 9.2 |  | 0.0100  ∗∗ |
| Steady-state activation | | 25 | | 21 | | | 19 | |
| Half potential V_1/2_ (mV) | -32.2  ± 1.1 |  | -29.7  ± 1.2 |  | 0.3397  *ns* | -30.8  ± 1.4 |  | 0.3397  *ns* |
| K slope factor | 6.4  ± 0.3 |  | 6.6  ± 0.2 |  | 0.6480  *ns* | 6.3  ± 0.3 |  | 0.6480  *ns* |
| Steady-state inactivation | | 25 | | 21 | | | 19 | |
| Half potential V_1/2_ (mV) | -80.7  ± 0.9 |  | -78.5  ± 1.2 |  | 0.2750  *ns* | -79.3  ± 1.0 |  | 0.2750  *ns* |
| K slope factor | -6.7  ± 0.2 |  | -7.0  ± 0.2 |  | 0.1626  *ns* | -7.5  ± 0.3 |  | 0.1626  *ns* |
| Recovery from inactivation |  | 25 | | 21 | |  | 19 | |
| Time constant τ fast (ms) | 8.3  ± 0.6 |  | 8.9  ± 0.9 |  | 0.1783  *ns* | 6.8  ± 0.8 |  | 0.1783  *ns* |
| Time constant τ slow (ms) | 97.4  ± 4.7 |  | 108.6  ± 10.7 |  | 0.6056  *ns* | 96.6  ± 12.2 |  | 0.6056  *ns* |

Data are represented in mean ± SEM and the replicate (n) represents the number of recorded cells. The significance was determined using the one-way ANOVA with Tukey’s multiple comparisons test. The *p* value represents the adjusted *p* value from Tukey’s multiple comparisons test when the initial one-way ANOVA is significant. Otherwise, the *p* value come from the one-way ANOVA when it is no significant. *ns*: no significant, ∗*p*<0.05, ∗∗*p*<0.01, ∗∗∗*p*<0.001 and ∗∗∗∗*p*<0.0001.

| Supplementary Table S4. Parameters of voltage-gated calcium channel biophysical properties. | | | | | | | | |
| --- | --- | --- | --- | --- | --- | --- | --- | --- |
| Parameters | **Conditions** | | | | | | | |
|  | CTRL | | DM1-1290 | | | DM1-1640 | | |
|  | Mean  ± SEM | n | Mean  ± SEM | n | *p* value | Mean  ± SEM | n | *p* value |
| VGCC channel properties | | | | | | | | |
| I_CaL_ currents | | 16 | | 29 | | | 17 | |
| Current density at 5 mV (pA/pF) | -14.0  ± 1.0 |  | -10.3  ± 0.9 |  | 0.0333  ∗ | -9.3  ± 1.3 |  | 0.0133  ∗ |
| Conductance G_max_ (pS) | 9.5  ± 1.1 |  | 12.4  ± 1.1 |  | 0.2260  *ns* | 12.8  ± 2.0 |  | 0.2260  *ns* |
| Steady-state activation | | 16 | | 29 | | | 17 | |
| Half potential V_1/2_ (mV) | -5.9  ± 0.6 |  | -6.8  ± 0.5 |  | 0.7384  *ns* | -9.7  ± 0.7 |  | 0.0007  ∗∗∗ |
| K slope factor | 6.5  ± 0.2 |  | 6.6  ± 0.2 |  | 0.9600  *ns* | 6.6  ± 0.4 |  | 0.9600  *ns* |
| Steady-state inactivation | | 16 | | 29 | | | 17 | |
| Half potential V_1/2_ (mV) | -29.8  ± 0.8 |  | -32.8  ± 0.6 |  | 0.0104  ∗ | -35.1  ± 0.8 |  | <0.0001  ∗∗∗∗ |
| K slope factor | -5.4  ± 0.2 |  | -5.0  ± 0.2 |  | 0.1672  *ns* | -5.6  ± 0.4 |  | 0.1672  *ns* |

Data are represented in mean ± SEM and the replicate (n) represents the number of recorded cells. The significance was determined using the one-way ANOVA with Tukey’s multiple comparisons test. The *p* value represents the adjusted *p* value from Tukey’s multiple comparisons test when the initial one-way ANOVA is significant. Otherwise, the *p* value come from the one-way ANOVA when it is no significant. *ns*: no significant, ∗*p*<0.05, ∗∗*p*<0.01, ∗∗∗*p*<0.001 and ∗∗∗∗*p*<0.0001. VGCC: voltage-gated calcium channel.

| Supplementary Table S5. Parameters of optical action potentials in vCMs and aCMs. | | | | | | | | |
| --- | --- | --- | --- | --- | --- | --- | --- | --- |
| Parameters | **Conditions** | | | | | | | |
|  | CTRL | | DM1-1290 | | | DM1-1640 | | |
|  | Mean  ± SEM | n | Mean  ± SEM | n | *p* value | Mean  ± SEM | n | *p* value |
| Optical action potentials (APs) in vCM monolayers | | | | | | | | |
| Activation map | | 12 | | 7 | | | 8 | |
| Conduction velocity CV (cm/s) | 9.4  ± 0.6 |  | 3.0  ± 0.5 |  | <0.0001  ∗∗∗∗ | 5.2  ± 0.4 |  | <0.0001  ∗∗∗∗ |
| Spontaneous beating frequency (Hz) | 0.5  ± 0.03 |  | 0.6  ± 0.08 |  | 0.3519  *ns* | 0.4  ± 0.06 |  | 0.3519  *ns* |
| AP durations | | 12 | | 7 | | | 8 | |
| At 50% of repolarization APD_50_ (ms) | 278.0  ± 13.2 |  | 312.9  ± 24.4 |  | 0.0231  ∗ | 381.3  ± 14.1 |  | <0.0001  ∗∗∗∗ |
| At 80% of repolarization APD_80_ (ms) | 327.9  ± 15.0 |  | 414.7  ± 25.1 |  | 0.0002  ∗∗∗ | 488.7  ± 13.9 |  | <0.0001  ∗∗∗∗ |
| Optical action potentials (APs) in aCM monolayers | | | | | | | | |
| Activation map | | 11 | | 10 | | | 11 | |
| Conduction velocity CV (cm/s) | 5.7  ± 0.7 |  | 2.7  ± 0.3 |  | 0.0002  ∗∗∗ | 2.9  ± 0.3 |  | 0.0005  ∗∗∗ |
| Spontaneous beating frequency (Hz) | 1.0  ± 0.1 |  | 1.1  ± 0.1 |  | 0.3709  *ns* | 1.3  ± 0.2 |  | 0.3709  *ns* |
| AP durations | | 11 | | 10 | | | 11 | |
| At 50% of repolarization APD_50_ (ms) | 144.3  ± 8.5 |  | 138.4  ± 9.7 |  | 0.8206  *ns* | 137.5  ± 6.7 |  | 0.8206  *ns* |
| At 80% of repolarization APD_80_ (ms) | 204.8  ± 11.4 |  | 184.6  ± 11.0 |  | 0.2269  *ns* | 182.1  ± 7.6 |  | 0.2269  *ns* |

Data are represented in mean ± SEM and the replicate (n) represents the number of recorded hiPSC-CM monolayers. The significance was determined using the one-way ANOVA with Tukey’s multiple comparisons test. The *p* value represents the adjusted *p* value from Tukey’s multiple comparisons test when the initial one-way ANOVA is significant. Otherwise, the *p* value come from the one-way ANOVA when it is no significant. *ns*: no significant, ∗*p*<0.05, ∗∗*p*<0.01, ∗∗∗*p*<0.001 and ∗∗∗∗*p*<0.0001. vCMs: ventricular-like cardiomyocytes derived from induced pluripotent stem cells, aCMs: atrial-like cardiomyocytes derived from induced pluripotent stem cells.

| Supplementary Table S6. Parameters of intracellular calcium transients in vCMs and aCMs. | | | | | | | | |
| --- | --- | --- | --- | --- | --- | --- | --- | --- |
| Parameters | **Conditions** | | | | | | | |
|  | CTRL | | DM1-1290 | | | DM1-1640 | | |
|  | Mean  ± SEM | n | Mean  ± SEM | n | *p* value | Mean  ± SEM | n | *p* value |
| Intracellular calcium transients (CT) in vCM monolayers | | | | | | | | |
| Activation map | | 12 | | 7 | | | 8 | |
| Ca^2+^ propagation vel. CaPV (cm/s) | 9.1  ± 0.6 |  | 2.5  ± 0.2 |  | <0.0001  ∗∗∗∗ | 4.8  ± 0.5 |  | <0.0001  ∗∗∗∗ |
| CT parameters | | 12 | | 7 | | | 8 | |
| Normalized amplitude (dF/F_0_) | 0.96  ± 0.6 |  | 0.94  ± 0.01 |  | 0.5528  *ns* | 0.93  ± 0.01 |  | 0.5528  *ns* |
| Time constant decay τ (ms) | 393.3  ± 7.1 |  | 380.4  ± 8.7 |  | 0.2301  *ns* | 409.9  ± 4.8 |  | 0.2301  *ns* |
| CT durations |  |  |  |  |  |  |  |  |
| At 50% of reuptake TD_50_ (ms) | 566.4  ± 8.5 |  | 520.0  ± 13.0 |  | 0.0025  ∗∗ | 636.4  ± 8.9 |  | <0.0001  ∗∗∗∗ |
| At 80% of reuptake TD_80_ (ms) | 761.1  ± 6.6 |  | 713.2  ± 9.3 |  | 0.0005  ∗∗∗ | 800.4  ± 8.2 |  | 0.0029  ∗∗ |
| Intracellular calcium transients (CT) in aCM monolayers | | | | | | | | |
| Activation map | | 11 | | 10 | | | 11 | |
| Ca^2+^ propagation vel. CaPV (cm/s) | 4.9  ± 0.6 |  | 2.4  ± 0.3 |  | 0.0008  ∗∗∗ | 2.4  ± 0.2 |  | 0.0006  ∗∗∗ |
| CT parameters |  |  |  |  |  |  |  |  |
| Normalized amplitude (dF/F_0_) | 0.86  ± 0.02 |  | 0.91  ± 0.02 |  | 0.0151  ∗ | 0.89  ± 0.02 |  | 0.1234  *ns* |
| Time constant decay τ (ms) | 211.6  ± 4.3 |  | 190.6  ± 7.5 |  | 0.0386  ∗ | 191.3  ± 5.5 |  | 0.0406  ∗ |
| CT durations | | 11 | | 10 | | | 11 | |
| At 50% of reuptake TD_50_ (ms) | 242.7  ± 5.6 |  | 213.7  ± 10.1 |  | 0.0456  ∗ | 212.9  ± 9.6 |  | 0.0345  ∗ |
| At 80% of reuptake TD_80_ (ms) | 350.3  ± 4.8 |  | 321.6  ± 10.1 |  | 0.0255  ∗ | 320.5  ± 9.6 |  | 0.0169  ∗ |

Data are represented in mean ± SEM and the replicate (n) represents the number of recorded hiPSC-CM monolayers. The significance was determined using the one-way ANOVA with Tukey’s multiple comparisons test. The *p* value represents the adjusted *p* value from Tukey’s multiple comparisons test when the initial one-way ANOVA is significant. Otherwise, the *p* value come from the one-way ANOVA when it is no significant. *ns*: no significant, ∗*p*<0.05, ∗∗*p*<0.01, ∗∗∗*p*<0.001 and ∗∗∗∗*p*<0.0001. vCMs: ventricular-like cardiomyocytes derived from induced pluripotent stem cells, aCMs: atrial-like cardiomyocytes derived from induced pluripotent stem cells.
